# Catabolic Ornithine Carbamoyltransferase Activity Facilitates Growth of Staphylococcus aureus in Defined Medium Lacking Glucose and Arginine

**DOI:** 10.1128/mbio.00395-22

**Published:** 2022-04-27

**Authors:** Itidal Reslane, Cortney R. Halsey, Amanda Stastny, Barbara J. Cabrera, Jongsam Ahn, Dhananjay Shinde, Madeline R. Galac, Margaret F. Sladek, Fareha Razvi, McKenzie K. Lehman, Kenneth W. Bayles, Vinai C. Thomas, Luke D. Handke, Paul D. Fey

**Affiliations:** a University of Nebraska Medical Center, Department of Pathology and Microbiology, Omaha, Nebraska, USA; b Multidrug-Resistant Organism Repository and Surveillance Network (MRSN), Walter Reed Army Institute of Research, Silver Spring, Maryland, USA; University of Illinois at Chicago

**Keywords:** arginine biosynthesis, *Staphylococcus aureus*, auxotrophy, transcriptional regulation

## Abstract

Previous studies have found that arginine biosynthesis in Staphylococcus aureus is repressed via carbon catabolite repression (CcpA), and proline is used as a precursor. Unexpectedly, however, robust growth of S. aureus is not observed in complete defined medium lacking both glucose and arginine (CDM-R). Mutants able to grow on agar-containing defined medium lacking arginine (CDM-R) were selected and found to contain mutations within *ahrC*, encoding the canonical arginine biosynthesis pathway repressor (AhrC), or single nucleotide polymorphisms (SNPs) upstream of the native arginine deiminase (ADI) operon *arcA1B1D1C1*. Reverse transcription-PCR (RT-PCR) studies found that mutations within *ccpA* or *ahrC* or SNPs identified upstream of *arcA1B1D1C1* increased the transcription of both *arcB1* and *argGH*, encoding ornithine carbamoyltransferase and argininosuccinate synthase/lyase, respectively, facilitating arginine biosynthesis. Furthermore, mutations within the AhrC homologue *argR2* facilitated robust growth within CDM-R. Complementation with *arcB1* or *arcA1B1D1C1*, but not *argGH*, rescued growth in CDM-R. Finally, supplementation of CDM-R with ornithine stimulated growth, as did mutations in genes (*proC* and *rocA*) that presumably increased the pyrroline-5-carboxylate and ornithine pools. Collectively, these data suggest that the transcriptional regulation of ornithine carbamoyltransferase and, in addition, the availability of intracellular ornithine pools regulate arginine biosynthesis in S. aureus in the absence of glucose. Surprisingly, ~50% of clinical S. aureus isolates were able to grow in CDM-R. These data suggest that S. aureus is selected to repress arginine biosynthesis in environments with or without glucose; however, mutants may be readily selected that facilitate arginine biosynthesis and growth in specific environments lacking arginine.

## INTRODUCTION

Staphylococcus aureus is a common cause of community-associated and hospital-acquired infections (1–3) and, due to the synthesis of a myriad of virulence factors, has the ability to infect multiple organ systems (4–7). However, to thrive in these unique niches, S. aureus must regulate its central metabolism to utilize the available carbon and nitrogen sources (8–11). Indeed, studies have shown that a functional glycolytic pathway is essential for S. aureus tissue invasion and overall virulence in a murine model of infection (12, 13). However, once an infection is established, it is predicted that S. aureus utilizes secondary carbon sources such as amino acids or peptides in niches (e.g., abscesses) where glucose is depleted due to the lack of vascularization and, in addition, rapid glucose consumption via phagocytic cells (14, 15). The milieu of a staphylococcal abscess is also predicted to be arginine depleted due to the upregulation of inducible nitric oxide synthase (iNOS) and arginase, both of which require arginine as a substrate (14, 16, 17). Indeed, a mutation in the arginine biosynthetic pathway impaired S. aureus kidney abscess persistence, indicating the importance of arginine biosynthesis in this niche (17). These observations suggest that the acquisition and consumption of peptides from host proteins may allow S. aureus to acquire the precursors required to support arginine biosynthesis in arginine-depleted environments such as an abscess. In support of this model, recent studies from our laboratory have revealed that S. aureus secretes proteases that are able to degrade collagen and encodes a peptide transporter (Opp3; FPR3757 locus SAUSA300_0887; GenBank accession number CP000255.1) that supports the growth of S. aureus on the degraded collagen peptides (14).

**FIG 1:**
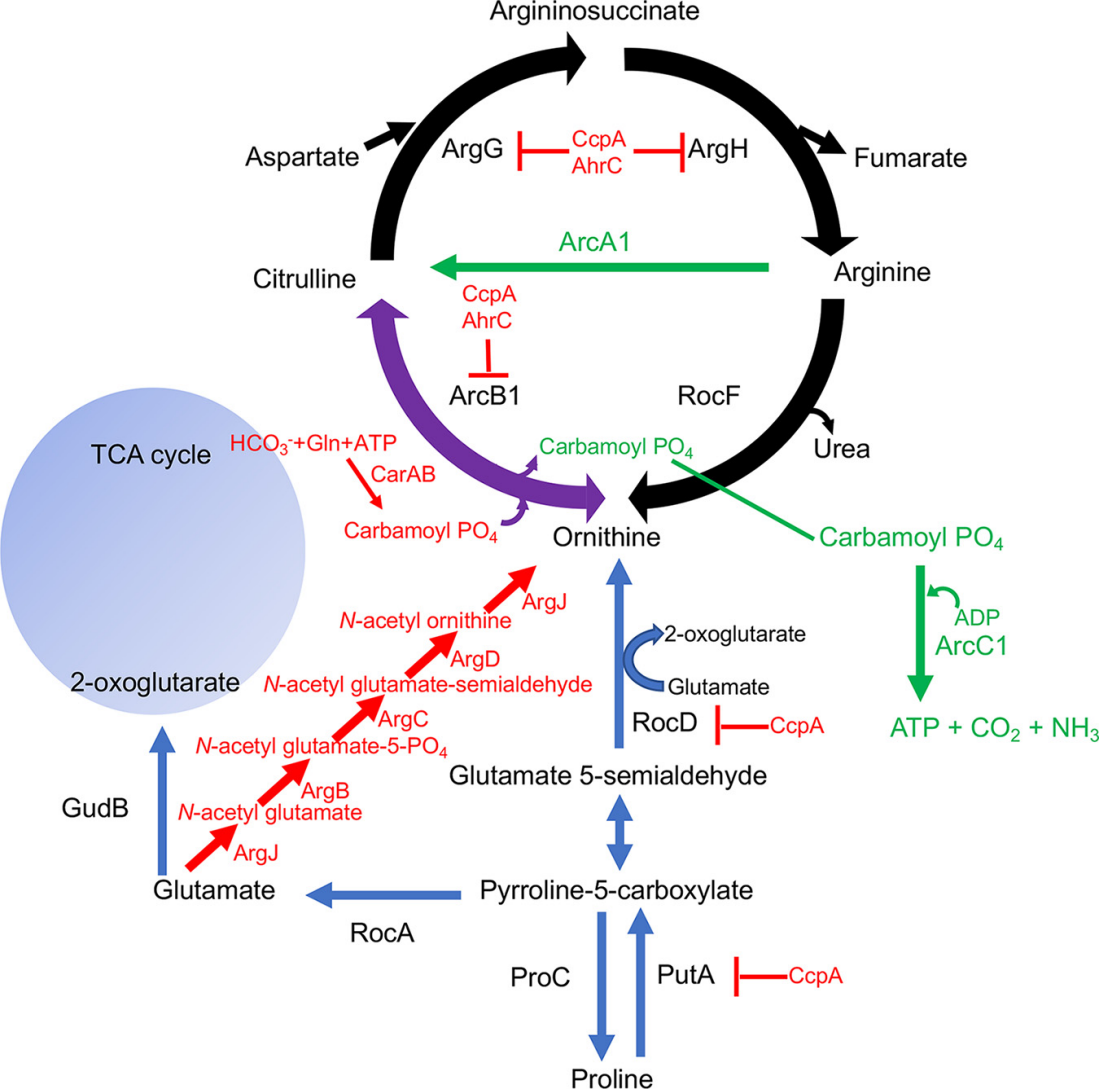
Arginine metabolic pathways in Staphylococcus aureus. Proline serves as a substrate for arginine biosynthesis via PutA, RocD, ArcB1, ArgG, and ArgH. The transcription of *putA*, *rocD*, *arcB1*, and *argGH* are repressed by CcpA, whereas *arcB1* and *argGH* are repressed by AhrC and CcpA. The canonical arginine biosynthetic pathway (in red) is repressed under the conditions studied in this article. The arginine deiminase pathway (shown in green) generates ATP, CO_2_, and NH_3_. All three pathways utilize ornithine carbamoyltransferase (ArcB1) (shown in purple). TCA, tricarboxylic acid.

It is well established that S. aureus displays multiple amino acid auxotrophies *in vitro*, including arginine, branched-chain amino acids, proline, valine, cysteine, and methionine (18–22). However, mutants can be isolated that are able to grow in media lacking any of these amino acids (19, 22), suggesting that all amino acid biosynthetic pathways are present but repressed during growth in standard laboratory media. S. aureus harbors the genes encoding the arginine biosynthetic pathway, *argJBCDFGH*, responsible for synthesizing arginine from glutamate (23). This canonical pathway is highly conserved and has been extensively studied in model prokaryotic systems, including Bacillus subtilis, Salmonella enterica serovar Typhimurium, and Escherichia coli (23–25). Studies from Nuxoll et al. documented that the growth of S. aureus in complete defined medium (CDM) containing 14 mM glucose but lacking arginine (CDMG-R) is dependent upon a *ccpA* mutation, thus alleviating carbon catabolite repression (CCR) (17). However, nuclear magnetic resonance (NMR) and genetic studies documented that the canonical arginine biosynthetic pathway using glutamate as a substrate was not utilized, but instead, proline served as the substrate (17). Altogether, these and other studies (14, 17, 26) documented that proline catabolism is repressed via carbon catabolite repression but can serve as a carbon source fueling glutamate synthesis when CcpA repression is alleviated, in addition to serving as a substrate for arginine biosynthesis. In agreement with previous observations, Halsey et al. found that *putA* and *argGH* transcription is upregulated in S. aureus JE2 *ccpA*::*tetL*, further confirming that arginine biosynthesis from proline is regulated by carbon catabolite repression (26) (Fig. 1).

Based on the above-mentioned observations, we predicted that robust growth of S. aureus would be observed in complete defined medium lacking glucose and arginine (CDM-R) due to the derepression of *putA* and *argGH* via the lack of CCR. Surprisingly, our current studies document that the growth of S. aureus in CDM-R is not robust and occurs only following ~16 h of incubation. Furthermore, we report here that mutations in several loci, including *ahrC*, encoding the canonical arginine biosynthetic pathway repressor, and single nucleotide polymorphisms (SNPs) in a region upstream of the arginine deiminase (ADI) operon (*arcA1B1D1C1*) facilitated robust growth in CDM-R. These mutations resulted in the upregulation of both *argGH* (argininosuccinate synthase/lyase) and *arcB1* (ornithine carbamoyltransferase), thus facilitating arginine biosynthesis via proline. Furthermore, we found that 53% of S. aureus clinical isolates could grow in CDM-R but not CDMG-R, suggesting that specific niches are present in the human host that may select for mutations resulting in arginine biosynthesis.

## RESULTS

### Growth of S. aureus in media lacking arginine.

**FIG 2:**
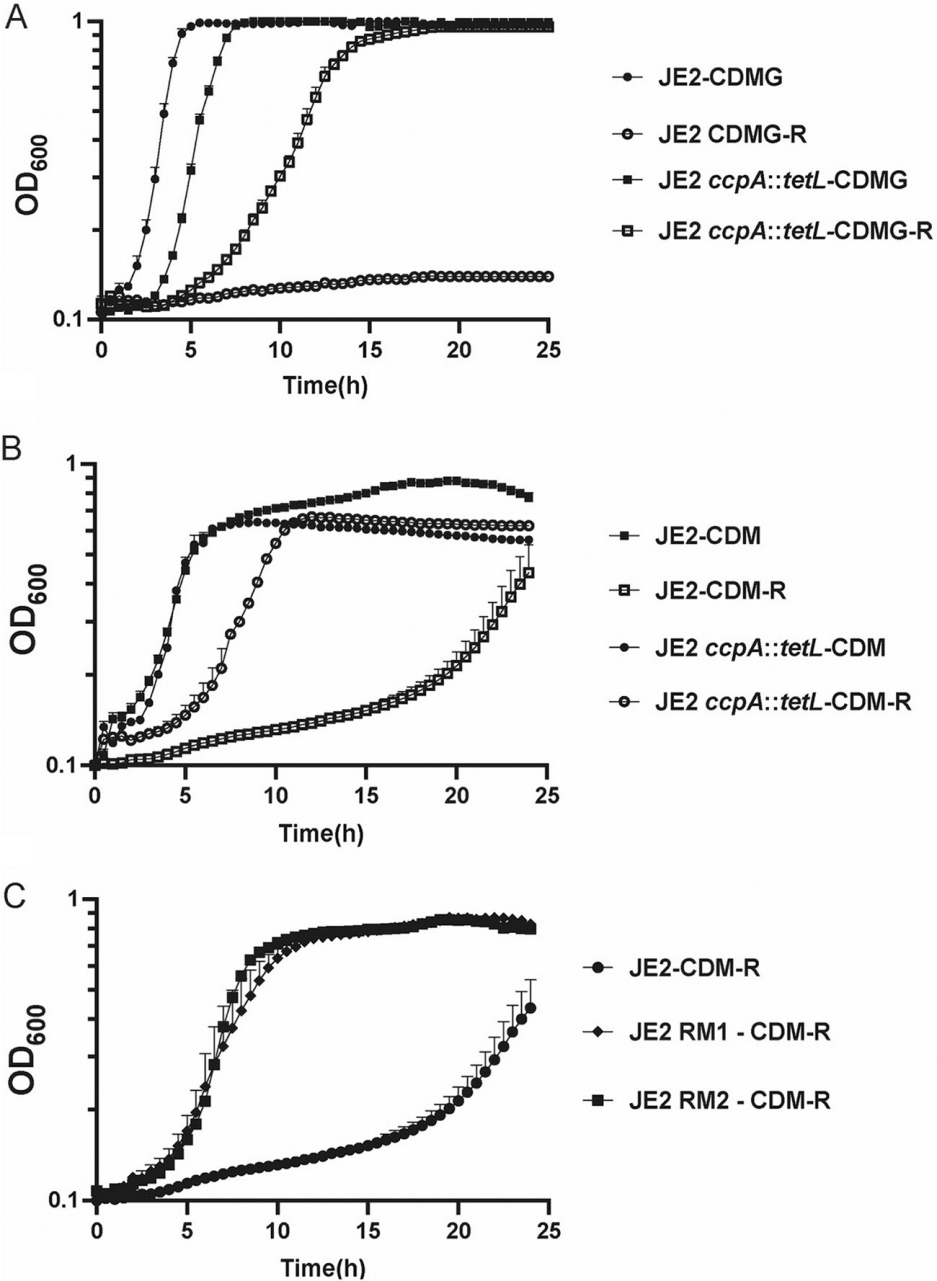
Mutations are selected in S. aureus facilitating growth in CDM-R. (A) Growth analysis of JE2 and JE2 *ccpA*::*tetL* in CDMG and CDMG-R. (B) Growth analysis of JE2 and JE2 *ccpA*::*tetL* in CDM and/or CDM-R. (C) Growth analysis of JE2 and two selected mutants able to grow on CDM-R agar (JE2 RM1 and RM2). Data represent results from three technical replicates per strain. Data are represented as means ± standard errors of the means (SEM).

Previous studies have demonstrated that CcpA and carbon catabolite repression (CCR) regulate arginine biosynthesis in S. aureus via the repression of *putA*, *rocD*, and *argGH* (17) (Fig. 1). Consistent with previous observations (17, 26), S. aureus growth in defined medium containing glucose but lacking arginine (CDMG-R) is dependent upon a mutation in *ccpA* (Fig. 2A). As CCR is alleviated when glucose is depleted from the medium, we hypothesized that S. aureus would grow in complete defined medium lacking both arginine and glucose (CDM-R). Unexpectedly, S. aureus JE2 exhibited an extended lag phase and a reduced growth rate in CDM-R; however, enhanced growth was consistently observed after 18 to 24 h of incubation (Fig. 2B). Since the lack of glucose in the media should alleviate CcpA-mediated repression, we were surprised to find that a *ccpA* mutation rescued the growth of JE2 in CDM-R similar to that observed in CDMG-R (Fig. 2A and B). These data suggest that CcpA is repressing arginine biosynthesis in a glucose-independent manner. To identify other loci that may regulate arginine biosynthesis, S. aureus mutants with the ability to grow robustly in the absence of arginine were selected by plating approximately 10^9^ CFU of S. aureus JE2 on CDM-R agar, and two colonies were selected for further study. As shown in Fig. 2C, these JE2 isolates (JE2 RM1 and RM2) exhibited rapid growth in CDM-R broth compared to wild-type (WT) JE2. Taken together, our results suggested that S. aureus does not grow robustly in the absence of arginine even when glucose is not present in the medium; however, growth can be selected via the selection of compensatory mutations.

### Mutations in *ahrC* and SNPs within the *arcA1B1D1C1* upstream region mediate robust growth of S. aureus in CDM-R.

Whole-genome sequencing was performed on five independently isolated JE2 isolates that were able to grow on CDM-R agar (see Table S1 in the supplemental material). Two isolates contained amino acid substitutions in the *ahrC* gene, encoding the arginine repressor AhrC. In B. subtilis, when bound to arginine, AhrC functions to repress the biosynthesis of arginine via binding to the *argCAEBD* promoter region, thus repressing the canonical arginine biosynthetic pathway (27, 28). In one of these mutants (mutant 17), JE2 *ahrC*_C124F_, a cysteine residue within the arginine binding pocket that forms hydrogen bonds with the arginine corepressor in the B. subtilis orthologue of AhrC was replaced with phenylalanine (27) (Fig. S1). In a second mutant (mutant 21), JE2 *ahrC*_K4N_, a lysine residue that has been shown to participate in DNA binding by B. subtilis AhrC was replaced by asparagine (Fig. S1) (27).

10.1128/mbio.00395-22.1 FIG S1 Alignment of the JE2 AhrC WT protein sequence (FPR3757) against the AhrC protein sequence of the isolated mutants JE2 *ahrC*_C124F_ and JE2 *ahrC*_K4N_. Blue amino acids represent the substituted residues in JE2 *ahrC*_C124F_ and JE2 *ahrC*_K4N_. Download FIG S1, PDF file, 0.01 MB. Copyright © 2022 Reslane et al. 2022 Reslane et al. https://creativecommons.org/licenses/by/4.0/ This content is distributed under the terms of the Creative Commons Attribution 4.0 International license.

10.1128/mbio.00395-22.8 TABLE S1 Identified mutations in JE2 isolates able to grow in CDM-R. Download Table S1, DOCX file, 0.01 MB. Copyright © 2022 Reslane et al. 2022 Reslane et al. https://creativecommons.org/licenses/by/4.0/ This content is distributed under the terms of the Creative Commons Attribution 4.0 International license.

**FIG 3:**
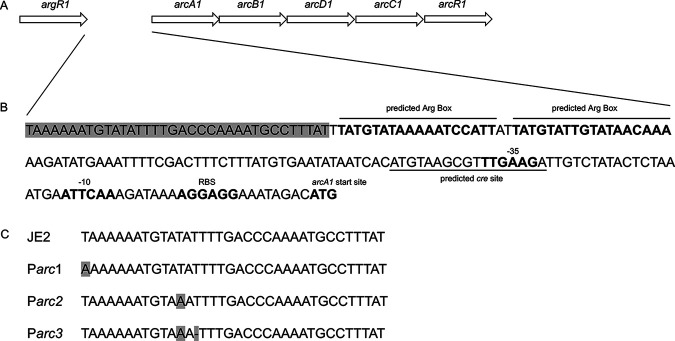
Single nucleotide polymorphisms (SNPs) identified upstream of the *arcA1B1D1C1* ATG start site. (A) Schematic representation of the S. aureus JE2 native arginine deiminase operon (*arcA1B1D1C1R1*) and the upstream native *argR1*. (B) Nucleotide sequence of the native *arc* operon region located upstream of the arginine deiminase (*arcA*) start site. The sequence in light gray represents the region where SNPs were detected. Predicted Arg boxes and *cre* site are underlined. RBS, ribosome binding site. (C) Identification of P*arc1*, P*arc2*, and P*arc3* SNPs (noted in the light gray region in panel B) compared to WT JE2. The base pair changes are shown in light gray. (Adapted from reference 64.)

In the remaining three isolates, single nucleotide polymorphisms (SNPs) were identified at different sites located upstream of the ATG start site of the *arcA1* gene (located in the native arginine deiminase operon [23] and not the arginine catabolic mobile element [ACME] arginine deiminase operon [29, 30]). These SNPs were located upstream of the proposed ARG boxes or the predicted *cre* site, both of which were found upstream of the *arcA1* ATG start site (Fig. 3). Mutants in this class were grouped according to the mutation position and are annotated as P*arc1*, P*arc2*, and P*arc3*. The organization of the *arcA1B1D1C1* operon, the predicted *arcA1B1D1C1* regulatory sequence, and the identified mutations are illustrated in Fig. 3.

### Arginine biosynthesis is dependent upon proline in JE2 *ahrC*_C124F_, JE2 *ahrC*_K4N_, and P*arc1*, P*arc2*, and P*arc3* mutants.

**FIG 4:**
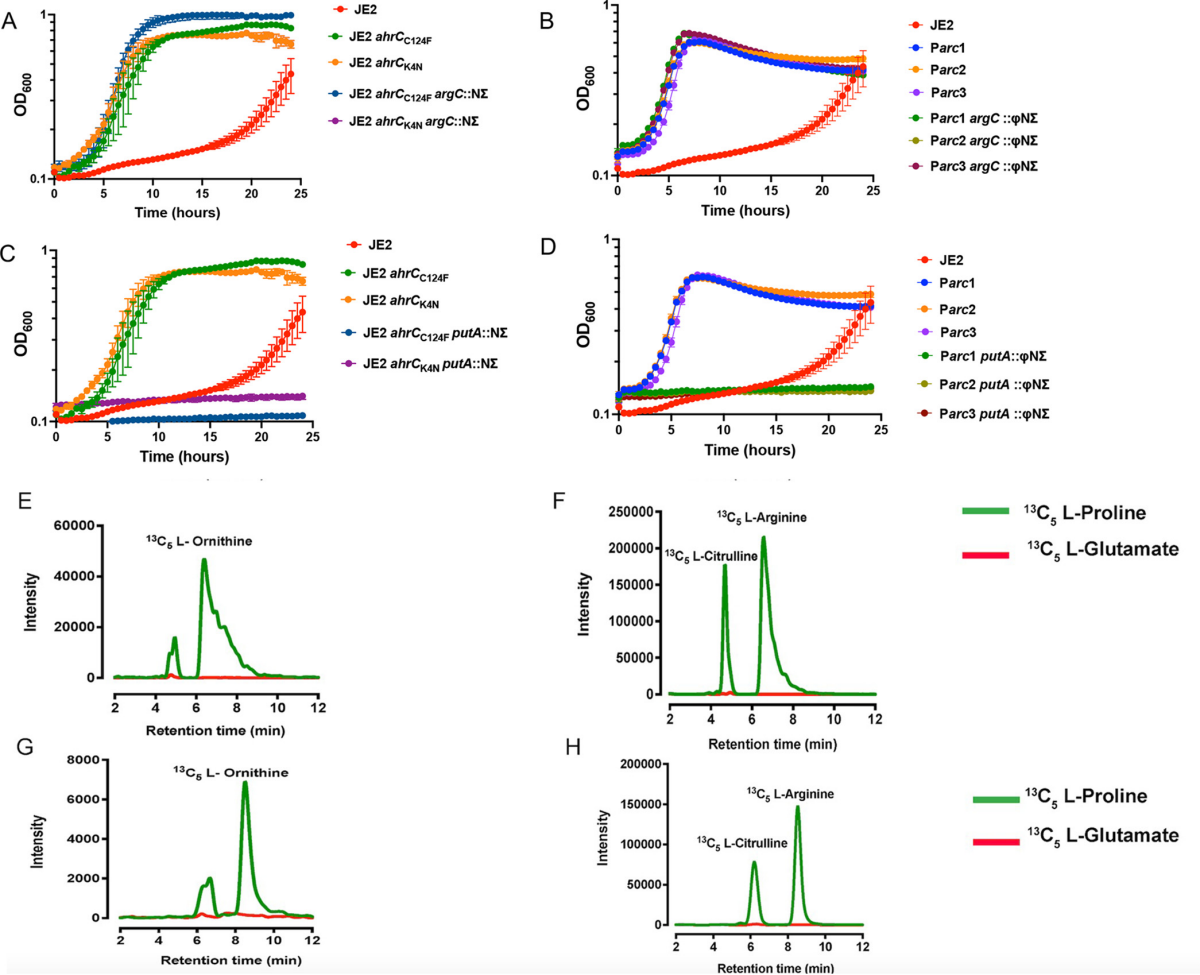
Proline serves as the substrate for arginine biosynthesis in *ahrC* and P*arc* mutants. (A and C) CDM-R growth analysis of JE2 *ahrC*_C124F_ and JE2 *ahrC*_K4N_ with *bursa aurealis* transposon insertions in *putA* (A) and *argC* (C). (B and D) CDM-R growth analysis of JE2 P*arc1*, P*arc2*, and P*arc3* with *bursa aurealis* transposon insertions in *putA* (B) and *argC* (D). Data represent results from three technical replicates per strain. Data are represented as means ± SEM (*n* = 3). (E to H) Liquid chromatography-tandem mass spectrometry (LC-MS/MS) analysis of the JE2 *ahrC*_C124F_ mutant (E and F) and the JE2 P*arc1* mutant (G and H) grown in CDM-R in the presence of either [^13^C_5_]proline or [^13^C_5_]glutamate. Data represent mean peak values from three biological replicates.

S. aureus harbors the alternative proline catabolic pathway as well as the canonical glutamate pathway to synthesize arginine (Fig. 1). To investigate which metabolic pathway contributed to the growth of JE2 *ahrC*_C124F_, JE2 *ahrC*_K4N_, and the P*arc* mutants in CDM-R, *bursa aurealis* transposon insertions within *putA* and *argC* from the Nebraska Transposon Mutant Library (31) were transduced into each strain. Our results demonstrated that growth in CDM-R was dependent upon PutA, but not ArgC, in JE2 *ahrC*_C124F_, JE2 *ahrC*_K4N_, and the P*arc* mutants, suggesting that proline was the precursor for arginine biosynthesis in all strains tested (Fig. 4A to D). Consistent with these observations, liquid chromatography-tandem mass spectrometry (LC-MS/MS) analysis showed that JE2 *ahrC*_C124F_ (Fig. 4E and F) and JE2 P*arc1* (Fig. 4G and H) grown in the presence of ^13^C_5_-labeled proline, but not ^13^C_5_-labeled glutamate, accumulated ^13^C_5_-labeled intracellular arginine, citrulline, and ornithine. Overall, our findings indicate that *ahrC* mutations and P*arc* SNPs mediate arginine biosynthesis via the proline catabolic pathway instead of the canonical glutamate pathway, which appears to be inactive under the conditions studied.

### AhrC negatively regulates arginine biosynthesis in S. aureus.

**FIG 5:**
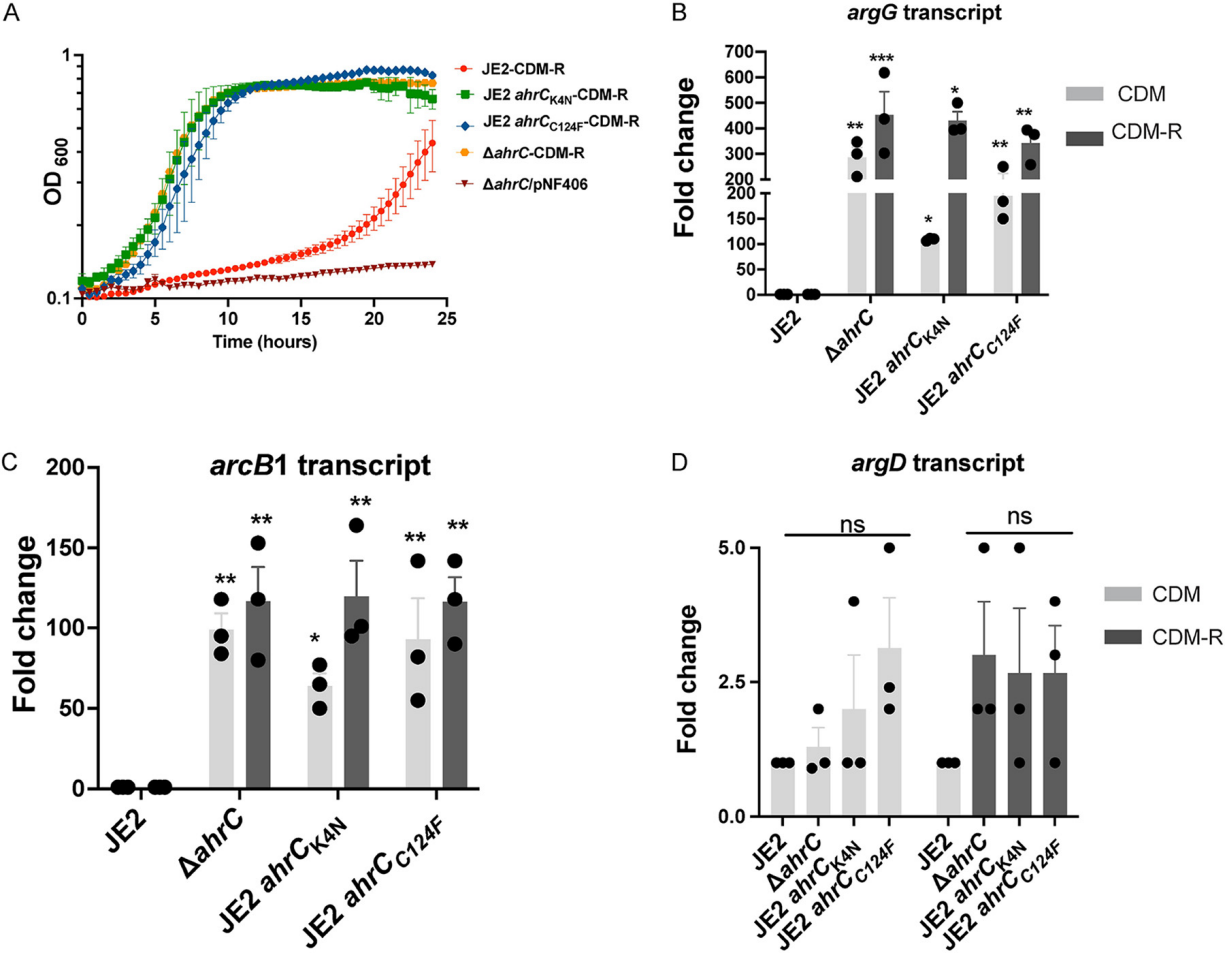
AhrC represses *argGH* and *arcB1* transcription. (A) Growth analysis of JE2, JE2 Δ*ahrC*, JE2 *ahrC*_C124F_, and JE2 *ahrC*_K4N_ in CDM-R and JE2 Δ*ahrC*/pNF406 (*ahrC* complement) in CDM-R. Data represent results from three technical replicates per strain. Data are represented as means ± SEM. (B and C) qRT-PCR assessing the transcripts of *argG* (B) and *arcB1* (C) in JE2 Δ*ahrC* and *ahrC* mutants in both CDM (light gray) and CDM-R (dark gray). (D) Expression of *argD* in JE2 Δ*ahrC* and *ahrC* mutants in CDM (light gray) and CDM-R (dark gray). Results are representative of data from three independent biological replicates performed with two technical replicates in each experiment. Error bars show means ± SEM (*n* = 3). Statistical significance was assessed using one-way ANOVA followed by Dunnett’s posttest. Asterisks indicate significant differences between WT JE2 and *ahrC* mutants. *, *P < *0.05; **, *P < *0.01; ***, *P < *0.001; ns, no significance.

Since our findings indicate that a mutation in *ahrC* facilitates the growth of S. aureus in CDM-R, a markerless Δ*ahrC* allelic replacement mutant was constructed to confirm these observations. Indeed, the growth of JE2 Δ*ahrC* phenocopied those of JE2 *ahrC*_C124F_ and JE2 *ahrC*_K4N_ in CDM-R (Fig. 5A). The introduction of the *ahrC* complementation plasmid pNF406 abrogated the growth of JE2 Δ*ahrC* in CDM-R (Fig. 5A). To further understand whether AhrC transcriptionally regulated a gene in the proline arginine biosynthetic pathway, the expression of *putA*, *rocD*, *arcB1*, and *argG* (Fig. 1) was assessed by quantitative reverse transcription-PCR (qRT-PCR) in JE2 *ahrC*_C124F_, JE2 *ahrC*_K4N_, and JE2 Δ*ahrC* grown in CDM and CDM-R and compared to that in wild-type JE2 in CDM. In addition, since S. aureus encodes three ornithine carbamoyltransferases (*arcB1*, *arcB2*, and *argF*), the transcription of *arcB2* and *argF* was determined. Furthermore, the transcription of *argD*, encoding acetylornithine aminotransferase (Fig. 1) functioning in the canonical arginine biosynthetic pathway, and *rocF*, encoding arginase (Fig. 1), were also assessed. The results demonstrated no change in the transcription of *putA*, *arcB2*, *argF*, *rocF*, and *rocD* (Fig. S2 and S3). However, significant upregulation of *argG* (Fig. 5B) and *arcB1* (Fig. 5C) was observed in JE2 Δ*ahrC*, JE2 *ahrC*_C124F_, and JE2 *ahrC*_K4N_ in both CDM and CDM-R. Furthermore, it is well characterized that AhrC represses the *argDCJB* operon in B. subtilis (32); however, in agreement with our genetic and LC-MS/MS data (Fig. 4), no significant change in *argD* expression in both CDM and CDM-R was noted for all *ahrC* mutants tested (Fig. 5D).

10.1128/mbio.00395-22.2 FIG S2 Quantitative reverse transcription-PCR (qRT-PCR) assessing the expression of *putA* (A and B) and *arcB2* (C and D) in JE2 *ahrC* mutants in both CDM and CDM-R. Data are representative of results from three independent biological replicates performed with two technical replicates in each experiment. Error bars show means ± SEM (*n* = 3). Statistical significance was assessed using one-way ANOVA followed by Dunnett’s posttest. None of the comparisons are significant. Download FIG S2, PDF file, 1.0 MB. Copyright © 2022 Reslane et al. 2022 Reslane et al. https://creativecommons.org/licenses/by/4.0/ This content is distributed under the terms of the Creative Commons Attribution 4.0 International license.

10.1128/mbio.00395-22.3 FIG S3 Quantitative reverse transcription-PCR (qRT-PCR) assessing the expression of *rocF* (A and B), *rocD* (C and D), and *argF* (E and F) in JE2 *ahrC* mutants in both CDM and CDM-R. Data are representative of results from three independent biological replicates performed with two technical replicates in each experiment. Error bars show means ± SEM (*n* = 3). Statistical significance was assessed using one-way ANOVA followed by Dunnett’s posttest. None of the comparisons are significant. Download FIG S3, PDF file, 0.7 MB. Copyright © 2022 Reslane et al. 2022 Reslane et al. https://creativecommons.org/licenses/by/4.0/ This content is distributed under the terms of the Creative Commons Attribution 4.0 International license.

### CcpA represses arginine biosynthesis in the absence of glucose.

**FIG 6:**
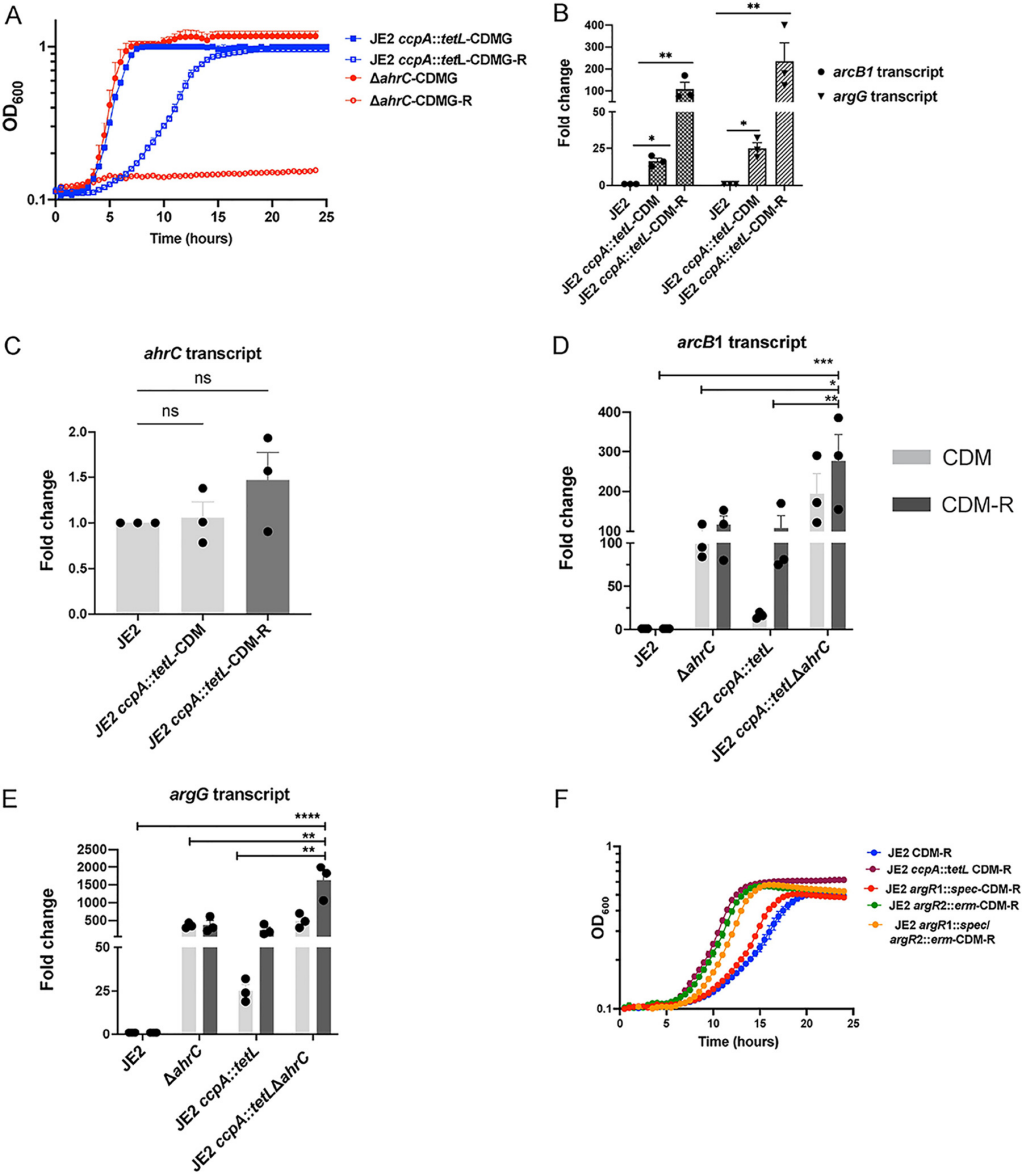
CcpA represses *argGH* and *arcB1* transcription during growth in CDM-R. (A) Growth analysis of JE2 Δ*ahrC* and JE2 *ccpA*::*tetL* in CDMG and CDMG-R. Data represent results from three technical replicates per strain. Data are represented as means ± SEM. (B) qRT-PCR assessing the transcripts of *arcB1* and *argG* in JE2 *ccpA*::*tetL* in both CDM (light gray) and CDM-R (dark gray). Statistical significance was assessed using one-way ANOVA followed by Dunnett’s posttest. Asterisks indicate significant differences between WT JE2 and JE2 *ccpA*::*tetL* (grown in CDM and CDM-R). (C) Expression of *ahrC* in JE2 *ccpA*::*tetL* in CDM and CDM-R compared to JE2. Statistical significance was assessed using one-way ANOVA followed by Dunnett’s posttest. (D and E) Expression of *arcB1* (D) and *argG* (E) in JE2 *ccpA*::*tetL* Δ*ahrC* in both CDM (light gray) and CDM-R (dark gray). Data are representative of results from three independent biological replicates performed with two technical replicates in each experiment. Error bars show means ± SEM (*n* = 3). Statistical significance was assessed using one-way ANOVA followed by Dunnett’s posttest and Tukey’s posttest. Significance was assessed between JE2 *ccpA*::*tetL* Δ*ahrC* (grown in CDM-R) and JE2 (grown in CDM), JE2 *ccpA*::*tetL* (grown in CDM-R), and JE2 Δ*ahrC* (grown in CDM-R). (F) Growth of JE2, JE2 *argR1*, JE2 *argR2*, JE2 *argR1 argR2*, and JE2 *ccpA* in CDM-R. *, *P < *0.05; **, *P < *0.01; ***, *P < *0.001; ****, *P < *0.001; ns, no significance.

Previous studies have documented that the growth of S. aureus in CDMG-R is dependent upon a mutation in *ccpA* as CcpA represses the transcription of *putA*, *rocD*, and *argGH* (17, 26, 33). Indeed, a mutation in *ahrC* does not facilitate the growth of JE2 in CDMG-R (Fig. 6A), presumably due to the repression of both *putA* and *rocD* via CcpA. In addition, previous studies document that derepression of both *putA* and *rocD* occurs during growth in media lacking glucose, such as CDM (26). Therefore, since growth in a medium lacking glucose should relieve CcpA-dependent repression, we were surprised to find that a mutation in *ccpA* rescued the growth of S. aureus in CDM-R (Fig. 2B). These results suggest that CcpA represses arginine biosynthesis in a glucose-independent manner and presumably at a locus different from *putA* or *rocD*. To more fully understand CcpA-dependent repression during growth in CDM and CDM-R, we quantified the expression of *argGH* and *arcB1* in JE2 *ccpA*::*tetL*. Similar to the Δ*ahrC* mutant, we observed significant upregulation of *arcB1* and *argG* (Fig. 6B) in JE2 *ccpA*::*tetL* when grown in both CDM and CDM-R. Interestingly, enhanced transcription of both *arcB1* and *argG* was noted in the *ccpA* mutant during growth in CDM-R compared to CDM. However, it is difficult to fully interpret these data as CDM-R transcript data were compared against JE2 growth in CDM and not CDM-R, due to the lack of robust growth in this broth. Taken together, these data indicate that both AhrC and CcpA regulate *arcB1* and *argGH* during growth in both CDM and CDM-R and that CcpA mediates repression in a glucose-independent manner during growth in CDM-R.

### AhrC and CcpA regulate arginine biosynthesis independently.

We further hypothesized that the increased expression of *argGH* and *arcB1* in JE2 *ccpA*::*tetL* during growth in CDM-R was because *ahrC* transcription was CcpA dependent. However, no significant change in *ahrC* expression was noted in JE2 *ccpA*::*tetL* in both CDM and CDM-R compared to WT JE2 (Fig. 6C), indicating that CcpA functions to repress arginine biosynthesis independently of AhrC.

To determine the contribution of both AhrC and CcpA to the regulation of *argGH* and *arcB1*, a JE2 *ccpA*::*tetL* Δ*ahrC* mutant was constructed, and the expression of *argGH* and *arcB1* was assessed in CDM and CDM-R. A significant increase in the transcription of *arcB1* (Fig. 6D) and *argG* (Fig. 6E) was noted in JE2 *ccpA*::*tetL* Δ*ahrC* during growth in CDM-R in comparison to either JE2 *ccpA*::*tetL* or JE2 Δ*ahrC* alone during growth in CDM-R, suggesting that the loss of both CcpA and AhrC leads to the further derepression of *arcB1* and *argG*.

### Growth of JE2 *argR1* and JE2 *argR2* in CDM-R.

S. aureus JE2 carries two other ArgR homologues besides *ahrC* (SAUSA300_1469; FPR3757 genome; BioProject accession number PRJNA16313): *argR1* (SAUSA300_2571) and *argR2* (SAUSA300_0066). *argR1* is located just upstream of the native arginine deiminase operon (Fig. 3), whereas *argR2* is located upstream of the arginine deiminase operon located within the ACME pathogenicity island (34). Therefore, JE2 *argR1*, JE2 *argR2*, and JE2 *argR1 argR2* were constructed and grown in CDM-R. In comparison to JE2, no significant CDM-R growth phenotype was noted with JE2 *argR1* (Fig. 6F). However, surprisingly, robust CDM-R growth was noted with JE2 *argR2* and JE2 *argR1 argR2* in comparison to JE2 and JE2 *argR1* (Fig. 5B). These data suggest that ArgR2, acquired on the ACME pathogenicity island, but not the native ArgR1, functions to regulate growth in CDM-R, in addition to AhrC.

### P*arc* mutants have enhanced expression of *arcB1* and *argGH*.

**FIG 7:**
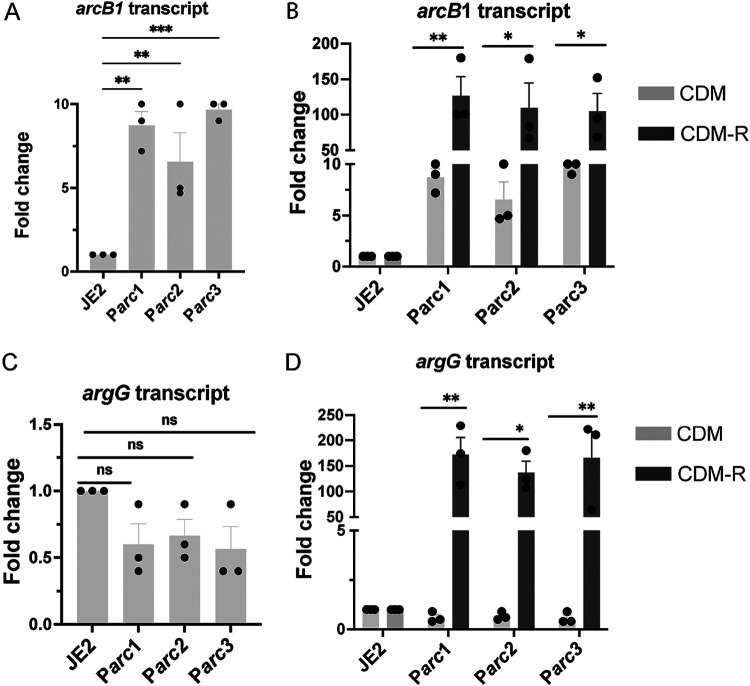
qRT-PCR assessing *arcB1* and *argGH* transcription in P*arc* mutants. Quantitative reverse transcription-PCR (qRT-PCR) was performed to assess the expression of *arcB1* (A and B) and *argG* (C and D) in JE2 P*arc* mutants in both CDM and CDM-R. Data are representative of results from three independent biological replicates performed with two technical replicates in each experiment. Error bars show means ± SEM (*n* = 3). Statistical significance was assessed using one-way ANOVA followed by Dunnett’s posttest (A and C) and Tukey’s posttest (B and D). Asterisks indicate significant differences between WT JE2 and P*arc* mutants (A and C) and between P*arc* mutants in CDM and CDM-R (B and D). *, *P < *0.05; **, *P < *0.01; ns, no significance.

Based on our findings that JE2 P*arc* mutants have enhanced growth in CDM-R, we sought to determine the impact of these SNPs on the transcription of arginine biosynthetic genes. Therefore, we performed qRT-PCR to assess the arginine biosynthetic gene expression (*putA*, *argD*, *arcB1*, *arcB2*, *argF*, *argG*, *rocF*, and *rocD*) of the P*arc* mutants grown in CDM and CDM-R. The level of gene expression was normalized against that of JE2 WT grown in CDM. As predicted, no change in the transcript levels of *putA*, *arcB2*, *argF*, *rocF*, and *rocD* was noted in all three of the mutants (Fig. S4 and S5). P*arc1*, P*arc2*, and P*arc3* exhibited a slight (~6-fold), yet significant, derepression of *arcB1* in CDM (Fig. 7A). In contrast, a substantial increase in *arcB1* transcription (~100-fold) was observed in CDM-R compared to CDM (Fig. 7B). Similar to *arcB1* expression, *argG* expression was strongly upregulated in CDM-R (Fig. 7D), while no change in expression was observed in CDM compared to WT JE2 (Fig. 7C and D).

10.1128/mbio.00395-22.4 FIG S4 Quantitative reverse transcription-PCR (qRT-PCR) assessing the expression of *putA* (A and B), *argD* (C and D), and *arcB2* (E and F) in JE2 P*arc* mutants in both CDM and CDM-R. Data are representative of results from three independent biological replicates performed with two technical replicates in each experiment. Error bars show means ± SEM (*n* = 3). Statistical significance was assessed using one-way ANOVA followed by Dunnett’s posttest. None of the comparisons are significant. Download FIG S4, PDF file, 0.7 MB. Copyright © 2022 Reslane et al. 2022 Reslane et al. https://creativecommons.org/licenses/by/4.0/ This content is distributed under the terms of the Creative Commons Attribution 4.0 International license.

10.1128/mbio.00395-22.5 FIG S5 Quantitative reverse transcription-PCR (qRT-PCR) assessing the expression of *rocF* (A and B), *rocD* (C and D), and *argF* (E and F) in JE2 P*arc* mutants in both CDM and CDM-R. Data are representative of results from three independent biological replicates performed with two technical replicates in each experiment. Error bars show means ± SEM (*n* = 3). Statistical significance was assessed using one-way ANOVA followed by Dunnett’s posttest. None of the comparisons are significant. Download FIG S5, PDF file, 0.7 MB. Copyright © 2022 Reslane et al. 2022 Reslane et al. https://creativecommons.org/licenses/by/4.0/ This content is distributed under the terms of the Creative Commons Attribution 4.0 International license.

### The anabolic activity of ornithine carbamoyltransferase (ArcB1) is essential for arginine biosynthesis in S. aureus.

**FIG 8:**
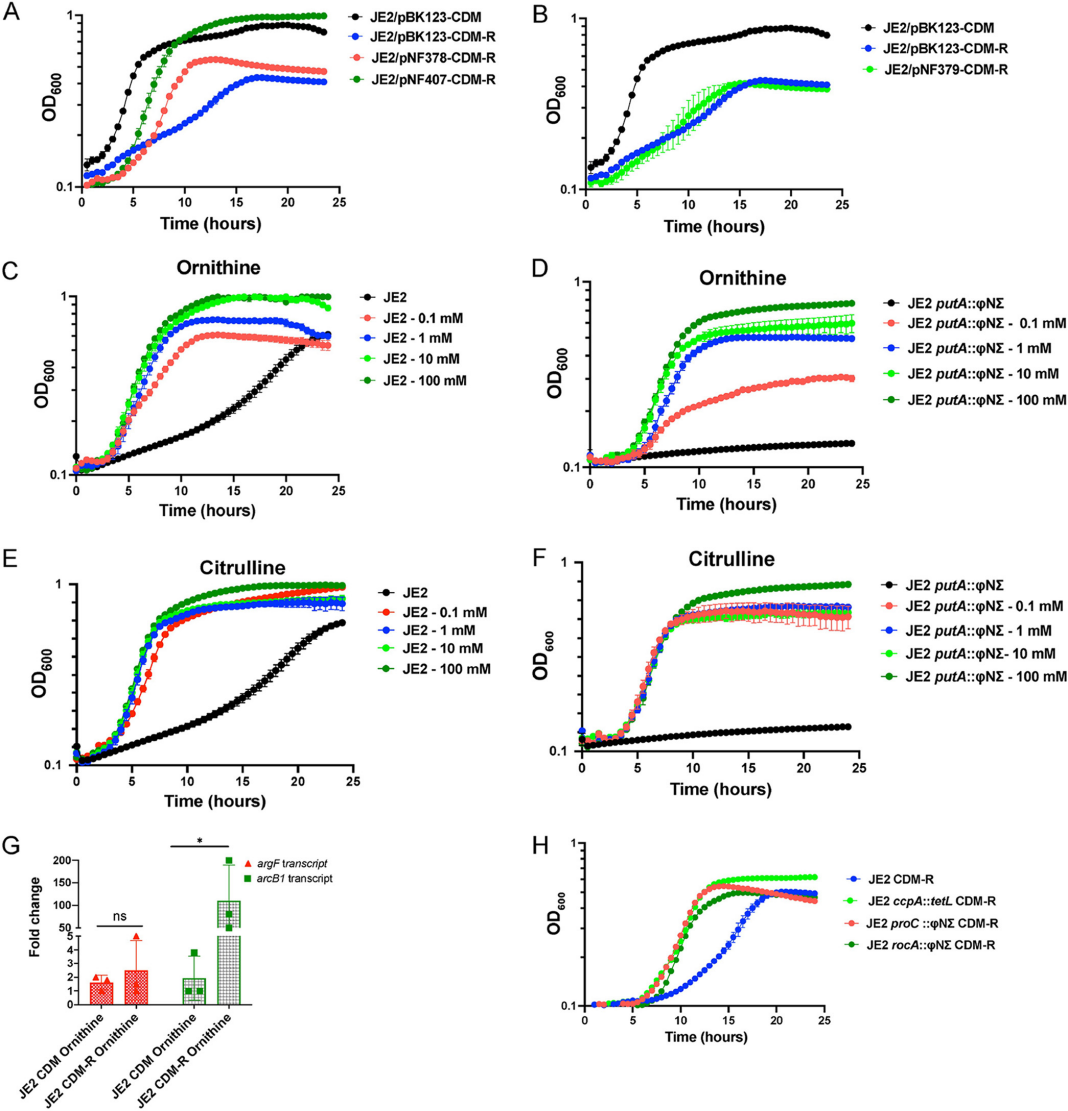
Complementation with *arcB1* or *arcA1B1D1C1* rescues the growth of JE2 in CDM-R. (A and B) Growth analysis of JE2/pNF378, JE2/pNF379, and JE2/pNF407 complementation plasmids harboring the *arcA1B1D1C1* operon, the *argGH* operon, and *arcB1*, respectively, in CDM-R. Growth of JE2/pBK123 (empty vector) in CDM and CDM-R was used as a control. (C to F) Growth of JE2 and JE2 *putA* in increasing concentrations of ornithine (C and D) and citrulline (E and F), respectively. (G) qRT-PCR assessing the transcripts of *argF* and *arcB1* in WT JE2 in both CDM with ornithine and CDM-R with ornithine. Data are representative of results from three independent biological replicates performed with two technical replicates in each experiment. Error bars show means ± SEM (*n* = 3). Statistical significance was assessed using one-way ANOVA followed by Dunnett’s posttest; *, *P < *0.05. (H) Growth of JE2, JE2 *ccpA*, JE2 *proC*, and JE2 *rocA* in CDM-R. Growth data in panels A to F and H represent results from three technical replicates per strain. Data are represented as means ± SEM.

As an SNP in the upstream region of *arcA1B1D1C1* alone facilitates growth in CDM-R, we surmised that the generation of citrulline may induce *argGH* transcription and, thus, that the critical block in arginine biosynthesis during growth in CDM-R is ornithine carbamoyltransferase (*arcB1*) transcription. To test this hypothesis, the expression plasmids pNF379, containing the *argGH* operon; pNF378, containing the *arcA1B1D1C1* operon; and pNF407, containing *arcB1*, were constructed with a cadmium-inducible promoter (P*cad*) and introduced into JE2, and growth was assessed in CDM-R. The introduction of both *arcB1* (pNF407) and *arcA1B1D1C1* (pNF378) rescued the growth of JE2 in CDM-R (Fig. 8A), although the induction of *arcB1* alone (pNF407) resulted in a higher growth yield. However, the introduction of *argGH* (pNF379) did not rescue the growth of JE2 in CDM-R (Fig. 8B). Furthermore, the addition of the substrate ornithine or the end product of ArcB1 catalysis, citrulline, rescues the growth of both JE2 and JE2 *putA* in CDM-R (Fig. 8C to F). Interestingly, qRT-PCR revealed that the addition of ornithine to CDM-R increased the expression of catabolic (ArcB1) and not anabolic (ArgF) (Fig. 8G) ornithine carbamoyltransferase. Therefore, we predicted that increasing the concentration of intracellular ornithine, by increasing the intracellular pyrroline-5-carboxylate (P5C) pools, would facilitate increased growth of S. aureus JE2 in CDM-R. To address this hypothesis, we grew JE2 *rocA* and JE2 *proC* in CDM-R, both of which have insertions in genes that encode proteins that utilize P5C as a substrate (Fig. 1). As predicted, mutations in both *rocA* and *proC* resulted in robust growth of JE2 in CDM-R (Fig. 8H). Collectively, these results provide evidence that enhanced transcription of *arcB1* and the subsequent anabolic activity of the catabolic ornithine carbamoyltransferase ArcB1 are crucial for growth in CDM-R. Furthermore, increasing the concentrations of ornithine, added either exogenously or via enhancing the intracellular P5C pools, also facilitates robust growth in CDM-R, presumably by the induction of *arcB1*. Finally, the addition of exogenous citrulline also mediates growth in CDM-R, suggesting that the presence of citrulline may induce *argGH* transcription.

### Growth of S. aureus clinical isolates in CDM-R.

To determine if clinical S. aureus isolates can grow in medium lacking arginine, 200 clinical isolates were selected and grown in both CDM-R and CDMG-R. One hundred isolates were collected from positive blood cultures (group 1), and an additional 100 isolates were collected from diverse sites, including skin and soft tissue, respiratory fluids, and bodily fluids (group 2). The BioProject accession number for these sequences is PRJNA731492 (35). Unexpectedly, the screen revealed that 54% and 53% of isolates from groups 1 and 2, respectively, demonstrated robust growth in CDM-R. However, none of the isolates able to grow in CDM-R could grow in CDMG-R (Fig. S6). Whole-genome sequencing of 40 isolates (20 per group) was performed to determine if SNPs could be identified in *ccpA*, *ahrC*, *proC*, *argR*, or the *arcA1B1D1C1* upstream region consistent with *in vitro*-selected mutations that facilitated arginine biosynthesis. Sequences from each clinical isolate were compared to the S. aureus USA300 FPR3757 genome sequence (GenBank accession number CP000255.1). Multilocus sequence typing (MLST) of the 40 sequenced isolates identified 15 sequence types (STs) (Table S3). Surprisingly, 16/40 isolates contained SNPs in the *arcA1B1D1C1* upstream region (compared to FPR3757), whereas 10/40 contained SNPs in both *ccpA* and the *arcA1B1D1C1* upstream region. Only two of the isolates contained SNPs in *ahrC*. Fourteen of the isolates had a similar *proC* V→I substitution at FPR3757 nucleotide 1605636, and 7 isolates had SNPs in *argR* homologues. Of the 44 isolates sequenced that had the ability to grow in CDM-R (Fig. S7), 16 did not have SNPs identified within the interrogated loci (Table S3). Taken together, these data indicate that arginine biosynthesis is selected within an *in vivo* environment. However, growth in the absence of arginine is still selected against when glucose is present.

10.1128/mbio.00395-22.6 FIG S6 Growth analysis in CDMG-R of S. aureus clinical isolates able to grow in CDM-R. Download FIG S6, PDF file, 1.6 MB. Copyright © 2022 Reslane et al. 2022 Reslane et al. https://creativecommons.org/licenses/by/4.0/ This content is distributed under the terms of the Creative Commons Attribution 4.0 International license.

10.1128/mbio.00395-22.7 FIG S7 Growth analysis of S. aureus clinical isolates able to grow in CDM-R. Download FIG S7, PDF file, 2.1 MB. Copyright © 2022 Reslane et al. 2022 Reslane et al. https://creativecommons.org/licenses/by/4.0/ This content is distributed under the terms of the Creative Commons Attribution 4.0 International license.

10.1128/mbio.00395-22.10 TABLE S3 SNPs identified in S. aureus clinical isolates able to grow in CDM-R. Download Table S3, XLSX file, 0.01 MB. Copyright © 2022 Reslane et al. 2022 Reslane et al. https://creativecommons.org/licenses/by/4.0/ This content is distributed under the terms of the Creative Commons Attribution 4.0 International license.

## DISCUSSION

Extensive studies of the arginine biosynthetic pathway have led to multiple significant shifts in thought, including genetic repression, the protein repressor, the regulon, gene duplication, the reactivation of silent genes through the generation of tandem repeats, promoter function, and evolutionary divergence (23, 36–41). Indeed, the biosynthesis of arginine from glutamate is a well-conserved pathway found in various bacteria, including Escherichia coli, B. subtilis, Pseudomonas aeruginosa, and S. aureus (23, 25, 42, 43). The regulation of genes involved in arginine biosynthesis differs among bacterial species (44–46); however, all arginine biosynthetic genes are transcriptionally regulated by ArgR-type transcriptional regulators (ArgR [E. coli/Salmonella] or AhrC [B. subtilis]) (27, 47–49). In the presence of arginine, ArgR/AhrC represses arginine biosynthetic genes by binding to DNA operator sites termed ARG boxes (50–53). Arginine acts as a corepressor for ArgR/AhrC, in which the binding of an arginine molecule induces a conformational change in the regulator, increasing the affinity for the ARG box (54). Therefore, in the absence of arginine, derepression of the ArgR/AhrC regulon occurs, facilitating biosynthesis using glutamate as a precursor (23, 49). Experimental evidence suggests that the ArgR/AhrC mechanism of regulation is conserved among Gram-positive, Gram-negative, and extremophilic bacteria (27, 28, 48, 50, 53, 55–57).

However, our data provide evidence that S. aureus synthesizes arginine via proline and not glutamate. Indeed, the growth of JE2 Δ*ahrC* in CDM-R was dependent upon *putA* and not *argC* (Fig. 4). Furthermore, our transcriptional analysis revealed that AhrC represses *argGH* and *arcB1*, with no significant change in the gene expression of *argDCJB* (Fig. 5; see also Fig. S2 in the supplemental material), indicating the presence of an additional transcriptional regulator that functions to repress the production of arginine using glutamate as a substrate. By the selection of mutants on CDM-R agar, we found that a mutation in AhrC facilitated robust growth in CDM-R. One of these mutations (C124F) was found in the domain that coordinates arginine binding, whereas the second mutation (K4N) was found in the DNA binding region (Fig. S1). This suggests that enough free arginine is present in the intracellular environment that binds AhrC when CDM-R is used as the growth medium or that AhrC can bind to specific cognate promoters in the absence of arginine. Further studies are required to determine if S. aureus AhrC functions in a manner different from that described for B. subtilis, E. coli, Lactococcus lactis, or P. aeruginosa (47, 53, 56, 58–61). In addition, we found that a mutation in *argR2*, but not *argR1*, facilitates the growth of S. aureus JE2 in CDM-R. This was unexpected since *argR2* is located within the acquired arginine catabolic mobile element (ACME) pathogenicity island, which is found primarily in ST8 USA300 isolates (29). Further studies are required to determine if ArgR2 functions to regulate arginine biosynthesis in a manner similar to that of AhrC or if they function independently of one another. We also found that a mutation in *ccpA* leads to robust growth in CDM-R by upregulating both *arcB1* and *argGH*. This was not expected as growth in media lacking glucose should alleviate CcpA-mediated repression. However, previous microarray studies using strain Newman identified CcpA-regulated genes in medium (LB) lacking glucose (62), including *arcC* and *argF*. Thus, it seems plausible that even though *putA* and *rocD* are derepressed during growth in CDM, CcpA could still repress *arcB1* transcription. In addition, the growth of S. aureus JE2 in CDM-R results in a long lag phase (~16 h), eventually leading to a growth yield of 0.45 (optical density at 600 nm [OD_600_]) by 24 h (Fig. 2B). At this point, it is unclear what the population of cells following 24 h of growth in CDM-R represents. Unfortunately, growth on solidified CDM or CDM-R is not consistent for unknown physiological reasons, and we are unsure if the CDM-R broth population primarily represents delayed induction of arginine biosynthesis via ArcB1/ArgGH and/or ornithine availability or possibly a selection of S. aureus cells with mutations in regulatory loci.

S. aureus converts arginine into ornithine, ammonia, carbon dioxide, as well as ATP via the arginine deiminase (ADI) pathway (Fig. 1). This pathway consists of three enzymes that catalyze the catabolism of arginine, arginine deiminase (ArcA), ornithine carbamoyltransferase (ArcB), and carbamate kinase (ArcC), encoded by the *arcA1B1D1C1* operon. The *arcD* gene encodes the arginine/ornithine antiporter allowing the exchange of one molecule of ornithine for each arginine molecule imported (23, 63). The S. aureus USA300 ST8 lineage possesses a second copy of the *arc* operon (*arcA2B2D2C2*) located on the ACME, a genomic island horizontally acquired from Staphylococcus epidermidis (34). In S. aureus SH1000, the native *arc* operon is induced under anaerobic growth conditions and was shown to be positively regulated by the transcription regulator ArcR (64). However, the ACME *arc* operon was reported to be constitutively expressed in USA300 under both aerobic and anaerobic conditions, promoting survival under acidic conditions (30). Our studies suggest that under the conditions tested, *arcB1* from the native arginine deiminase operon (*arcA1B1D1C1*) is repressed by AhrC during aerobic growth. A significant upregulation of ornithine carbamoyltransferase (*arcB1*), a potential surrogate for the entire *arc* operon, was noted in the selected JE2 *ahrC* mutants (Fig. 5). However, it is unclear if *arcB1* transcription is regulated independently from the *arcA1B1D1C1* operon. We surmise that the derepression of *arcB1* is mediated to fulfill an anabolic function (arginine biosynthesis) rather than a catabolic one. Indeed, the overexpression of *arcA1B1D1C1* facilitated the growth of JE2 when grown in CDM-R (Fig. 8A). One would presume that the upregulation of *arcB1* would also result in the upregulation of the ADI operon and, thus, the catabolism of arginine via arginine deiminase. The decreased growth yield observed when the entire *arc* operon was induced (JE2/pNF378) (Fig. 8A) in comparison to *arcB1* (JE2/pNF407) (Fig. 8A) may indicate that some of the arginine produced was catabolized via ArcA and the ADI pathway. Nevertheless, when *arcB1* was artificially induced (via pNF407), a growth yield of an OD_600_ of 1 was observed, similar to that of JE2 grown in CDM, indicating that the anabolic activity of ArcB1 is essential for growth when arginine is limiting.

S. aureus harbors two catabolic ornithine carbamoyltransferases, ArcB1 (native) and ArcB2 (ACME encoded), and a proposed anabolic ornithine carbamoyltransferase (ArgF). The catabolic enzymes typically convert citrulline into ornithine, facilitating catabolism via ADI, while the anabolic enzyme converts ornithine into citrulline, facilitating biosynthesis via the urea cycle (Fig. 1). We observed no change in the expression of *arcB2* and *argF* in all JE2 *ahrC* mutants (Fig. S2 and S3). In addition, accumulation of citrulline was observed in all the mutants tested via LC-MS/MS (Fig. 4F and H), indicating that ArcB1 is converting ornithine into citrulline, thus fulfilling the anabolic activity of ArgF. Furthermore, the addition of ornithine to CDM-R induces the expression of *arcB1* and not *argF* (Fig. 8). Based on the above-described results, we conclude that the low expression of *arcB1* contributes to the conditional arginine auxotrophy, and furthermore, S. aureus utilizes a catabolic instead of an anabolic enzyme to facilitate arginine biosynthesis via the urea cycle. Interestingly, studies of Streptococcus gordonii, which lacks an anabolic ornithine carbamoyltransferase, documented that *arcB* mutants were unable to grow in the absence of arginine under anaerobic conditions (65). Furthermore, in P. aeruginosa, an *argF* mutant defective in the anabolic ornithine carbamoyltransferase (ArgF) can grow in media lacking arginine after extended incubation, indicating that ArcB can compensate for ArgF activity (66). Although S. aureus is clearly utilizing ArcB1 to mediate arginine biosynthesis under the conditions tested, multiple kinetics investigations have shown that the anabolic reaction catalyzed by ArgF is highly efficient and thermodynamically favored (67, 68). Therefore, kinetic studies using S. aureus ArcB1, ArcB2, and ArgF are required to further address the function of ArgF. We hypothesize that ArgF has a higher affinity for ornithine than for ArcB1. As noted above, S. aureus heavily represses and tightly regulates arginine biosynthesis. Thus, utilizing the anabolic ArgF may interfere with adaptive mechanisms acquired by the pathogen to maintain conditional auxotrophy. Thus, we speculate that S. aureus has been selected to use the less thermodynamically favored catabolic enzyme to more tightly regulate the biosynthesis of arginine, although it is unclear why conditional auxotrophy may be favored in particular environments. Finally, since the addition of exogenous ornithine stimulates the growth of JE2 in CDM-R in addition to mutations in *rocA* and *proC*, it is possible that the conversion of P5C to glutamate via RocA (P5C dehydrogenase) or proline via P5C reductase (ProC) is thermodynamically favored over conversion to ornithine via RocD (ornithine aminotransferase). Further, since glutamate demand is high due to its use as a major carbon source during growth in CDM (26), it is also possible that RocD activity is limited due to the requirement of glutamate as an amino donor (Fig. 1). If so, this would result in low cellular ornithine pools, resulting in the poor growth yield observed in CDM-R. Indeed, previous NMR studies assessing the growth of S. aureus JE2 in CDM containing ^13^C-labeled proline noted significant quantities of labeled glutamate and small amounts of ornithine; no ^13^C-labeled citrulline or arginine was detected (26). These data suggest that the ornithine pool is indeed small during growth in CDM-R; in addition, the transcription of the catabolic ornithine carbamoyltransferase (*arcB1*) is repressed, further limiting citrulline synthesis. Finally, we were surprised to find that a *proC* mutation resulted in robust growth in CDM-R, similar to a *rocA* mutant. This suggests that ProC is active during PutA-dependent proline catabolism (Fig. 1). This appears to be a futile cycle; however, a requirement for ProC activity may be linked to the regulatory activity of or potential toxicity associated with intracellular concentrations of P5C.

During infection, S. aureus often encounters diverse environments requiring an adjustment of its central metabolism to rapidly changing carbon and nitrogen sources to maintain survival and persistence. Along with glucose, amino acids are an important carbon source for S. aureus growth and proliferation within the host (69). S. aureus encodes pathways required for the biosynthesis of all 20 amino acids, in addition to harboring transporters enabling it to acquire amino acids from the host or synthesize them *de novo* (70). Recent studies documented that S. aureus relies on aspartate biosynthesis for proliferation and survival during bone infection due to excess glutamate in infected tissues that inhibits aspartate acquisition (11). Likewise, S. aureus arginine biosynthesis promotes kidney abscess persistence (17). This indicates that spatial differences in the metabolic requirements for amino acid biosynthetic pathways exist during infection and suggests that S. aureus can selectively activate certain biosynthetic pathways while repressing others depending on the niches that it colonizes. Indeed, our clinical isolate screen suggests that the repression of arginine biosynthesis is advantageous to growth or survival within certain ill-defined niches but selected against in others, particularly in niches containing glucose. Although our genomic sequencing data need to be confirmed using genetic experimentation, it is interesting to note that many of our clinical isolates have SNPs in loci that confer the ability to grow in CDM-R.

It remains unclear what selective pressure governs the activation of the arginine biosynthetic pathway or why mutations are required to completely derepress these pathways. One possibility is that the selection of mutations provides fitness for S. aureus under certain stress conditions in which arginine might be serving as a signaling molecule/sensor. Previous studies documented the importance of arginine biosynthesis for multiple pathogens, including Mycobacterium tuberculosis, Listeria monocytogenes, and Mycobacterium bovis. Arginine biosynthetic genes were found to be upregulated and essential for the intracellular growth of L. monocytogenes (71). In addition, *de novo* arginine biosynthesis was shown to be highly important for the growth of M. tuberculosis, protecting the pathogen from DNA damage induced by reactive oxygen species (ROS) generation (72). Furthermore, l-arginine and l-ornithine supported the intracellular growth of M. bovis (73). Overall, it appears that arginine biosynthesis is linked to the virulence of multiple microorganisms, and it is an important component that can influence pathogen survival and persistence within the host cell. Finally, in B. subtilis, the transcriptional derepression of the arginine biosynthetic pathway by AhrC results in the activation of the pathway as well as the autorepression of AhrC itself (54). Therefore, AhrC may positively regulate several downstream effector molecules that are required for growth and survival; hence, transcriptional derepression might be deleterious for S. aureus. Note that most (63%) of the sequenced clinical isolates contained SNPs within the promoter region of *arcA1B1D1C1*, thus potentially directly upregulating *arcB1*, whereas only two sequenced isolates contained a mutation in *ahrC*.

In conclusion, our data suggest that the poor growth of S. aureus in CDM-R is blocked by reduced ornithine carbamoyltransferase (ArcB1) activity and that robust growth is mediated by either increasing the ArcB1 enzyme concentration or increasing the substrate concentration (ornithine). At least two regulators were identified (AhrC and CcpA) that function to repress *arcB1* transcription; mutations within these regulators resulted in increased *arcB1* transcription and subsequent arginine biosynthesis as assessed by growth studies and LC-MS/MS. Furthermore, we found that the addition of exogenous ornithine and citrulline facilitated robust growth in CDM-R, suggesting that elevated cytoplasmic ornithine or citrulline concentrations may induce *arcB1* and *argGH* transcription, respectively. Indeed, exogenous ornithine alone induced *arcB1* transcription. Finally, we found that presumably increasing the ornithine pool by diverting P5C away from glutamate or proline (*rocA* and *proC* mutations, respectively) also resulted in robust growth in CDM-R.

## MATERIALS AND METHODS

### Bacterial strains and culture conditions.

For examination of arginine auxotrophy among S. aureus clinical strains, 200 deidentified isolates were obtained from the Clinical Microbiology Laboratory at Nebraska Medicine. Additional S. aureus strains used in this study are listed in Table S2 in the supplemental material. Defined *bursa aurealis* transposon mutants were obtained from the Nebraska Transposon Mutant Library and backcrossed to JE2 using Φ11 (31). JE2 and S. aureus clinical strains were grown overnight in 5 mL tryptic soy broth (TSB) at 37°C with shaking at 250 rpm. Cultures grown overnight were washed with phosphate-buffered saline (PBS) twice and inoculated to an optical density at 600 nm (OD_600_) of 0.05. Growth analysis was performed in complete defined medium (CDM) and CDM-R with no glucose (74) added to a 96-well plate in an Infinite 200 Pro device (Tecan) at 37°C with shaking at 250 rpm. A total of 14 mM glucose was added when appropriate (CDMG). Plasmids constructed to express *arcA1BDC* (pNF378), *argGH* (pNF379), *arcB1* (pNF407), and *ahrC* (pNF406) utilized the pBK123 plasmid backbone derived from pCN51 (75). Each DNA fragment was amplified from JE2 by PCR using primers listed in Table S2. Double-stranded DNA (dsDNA) fragments with overlapping ends complementary to the vector were ligated into the SalI and XmaI sites of pBK123 using Gibson assembly (76, 77). The expression of *arcB1*, *argGH*, and *ahrC* was induced via a cadmium-inducible promoter (75). The completed constructs were electroporated into S. aureus RN4220 (78, 79), confirmed using primers noted in Table S2, and transduced into JE2 via Φ11 transduction (80). Growth analysis of JE2/pNF378, JE2/pNF379, JE2/pNF406, and JE2/pNF407 was conducted in a 96-well plate as described above except that the TSB culture grown overnight contained 5 μg/mL chloramphenicol, while CDM/CDM-R contained 1 μg/mL chloramphenicol and 100 nM cadmium chloride.

10.1128/mbio.00395-22.9 TABLE S2 Strains, primers, and probes used in this study. Download Table S2, DOCX file, 0.04 MB. Copyright © 2022 Reslane et al. 2022 Reslane et al. https://creativecommons.org/licenses/by/4.0/ This content is distributed under the terms of the Creative Commons Attribution 4.0 International license.

### qRT-PCR.

Cultures of S. aureus JE2, JE2 *ahrC*, JE2 Δ*ahrC*, and JE2 P*arc* mutants were grown overnight in 3 mL CDM at 37°C with shaking at 250 rpm. Cultures grown overnight were inoculated to an OD_600_ of 0.05 into 25 mL of CDM or CDM-R in a 250-mL flask (1:10 volume-to-flask ratio). Cells were grown aerobically (250 rpm) to exponential phase and collected at an OD_600_ of 0.4 to 0.8. Six milliliters of Qiagen RNA protect bacterial reagent was added to 3 mL of the collected culture. Cells were incubated for 5 min at room temperature and pelleted by centrifugation for 10 min at full speed. The pellet was resuspended in 185 μL of lysis buffer, followed by 15 μL of proteinase K. Samples were incubated on a rotating platform shaker at room temperature for 10 min and then resuspended in 700 μL RLT buffer containing 1% β-mercaptoethanol. Next, suspensions were transferred to lysing matrix B tubes (MP Biomedicals) and processed in an FP120 FastPrep cell disrupter (MP Biomedicals) for 45 s at a setting of 6.0. A total of 760 μL of the supernatant was transferred into a new tube containing 590 μL of 80% ethanol. The samples were then processed using an RNeasy minikit, according to the manufacturer’s instructions (Qiagen, Inc.). cDNA was generated using SuperScript IV Vilo master mix with ezDNase enzyme (Thermo Fisher Scientific) with 1 pg to 2.5 μg total RNA in each 10-μL reaction mixture. Reactions without reverse transcriptase were also performed for each RNA sample, and the samples were confirmed to be free of contaminating genomic DNA by PCR. All primers and probes used in this study are listed in Table S2. Primer-probe mixes (20×) containing 10 μM each primer and 4 μM labeled probe were prepared. All qRT-PCRs were performed with a total volume of 20 μL (10 μL TaqMan Fast advanced master mix [Thermo Fisher], 1 μL of 20× primer-probe mix, 4 μL of H_2_O, 5 μL of diluted template cDNA). Copy numbers of each transcript were determined against a standard curve performed with dilutions of plasmid clones carrying each gene target, and data were normalized against the geometric means for two reference genes, *gyrB* (81) and *tpiA* (82). Standards and diluted cDNA samples were assayed in duplicate with a QuantStudio 3 instrument (Thermo Fisher Scientific).

### Construction of JE2 Δ*ahrC*.

The pNF293 JE2 Δ*ahrC* allelic replacement construct was first created by the insertion of an 861-bp *ahrC* upstream PCR product using primers 1999 and 2000 (Table S2) into the EcoRI and BamHI sites of pUC19 (83). Second, a 755-bp *ahrC* downstream PCR product was amplified using primers 2211 and 2212 (Table S2) and ligated into the BamHI and PstI sites of the pUC19 polylinker. The temperature-sensitive pE194 derivative pROJ6448 was ligated into the PstI site of the plasmid (84). The completed construct, pNF293, was electroporated into the restriction-deficient S. aureus strain RN4220 (79). pNF293 was subsequently transduced into S. aureus JE2 using phage 80α as previously described (85). Allelic replacement was performed using previously described methods (86). Sequencing (Eurofins Genomics) using primers 2944 and 2945 (Table S2) confirmed the in-frame deletion of *ahrC* in JE2 Δ*ahrC*.

### Liquid chromatography-tandem mass spectrometry sample preparation.

Cultures of S. aureus JE2, JE2 *ahrC*, and JE2 P*arc* mutants were grown overnight in 3 mL CDM at 37°C with shaking at 250 rpm. A total of 1.5 mL of the culture grown overnight was centrifuged, and cell pellets were washed twice with 1 mL of a 0.85% saline solution and resuspended in 0.5 mL of CDM-R containing 1.3 mM ^13^C_5_-labeled proline or 1 mM ^13^C_5_-labeled glutamate. Next, cells were inoculated to an OD_600_ of 0.05 into 25 mL of CDM-R containing 1.3 mM ^13^C_5_-labeled proline or 1 mM ^13^C_5_-labeled glutamate in a 250-mL flask (1:10 volume-to-flask ratio), grown to exponential phase, and collected when an OD_600_ of 0.5 was reached. Ten OD_600_ units were harvested and transferred to a filter system, where they were washed twice with 5 mL of a cold and isotonic NaCl solution to guarantee the exact separation of intra- and extracellular metabolites. The filter was subsequently transferred to a 50-mL centrifuge tube containing 5 mL of an extraction solution consisting of 60% ethanol and 2 μM Br-ATP. The solution was hand mixed and alternatively vortexed 10 times to extract the cells from the filter. A total of 1.5 mL of the cell suspension was transferred to a homogenizer tube with 0.5 mL of glass beads. Each sample was aliquoted into 3 homogenizer tubes to avoid a high ratio of cell biomass to glass beads. Finally, cells were disrupted with 3 cycles in a Precellys homogenizer for 30 s at 6,800 rpm at 4°C. The homogenized mixture was centrifuged at 12,000 rpm for 10 min, and 4 mL of the supernatant and pool was collected. Three milliliters of the pooled sample was used for further analysis by liquid chromatography-tandem mass spectrometry (LC-MS/MS).

### LC-MS/MS separation and quantitation.

LC-MS/MS separation and quantitation were carried out using an XBridge amide 3.5-μm (2.1- by 100-mm) column procured from Waters. Mobile phase A contained 10 mM ammonium acetate and 10 mM ammonium hydroxide in water with 5% acetonitrile, whereas mobile phase B contained 100% acetonitrile. The flow rate was 0.4 mL/min, with a gradient mode of mobile phases. The column was maintained at 40°C. Detection of metabolites was carried out using the Qtrap 6500 system (Sciex) in multiple-reaction mode (MRM). All labeled metabolites, such as canonical and noncanonical amino acid standard mixtures, [^13^C_5_]arginine, [^13^C_5_]glutamate, and [^13^C_5_]proline, were purchased from Cambridge Isotopes, Inc.

### Genome sequencing and multilocus sequence typing.

DNA from JE2 strains was extracted using the DNeasy UltraClean microbial kit (Qiagen, Germantown, MD, USA), and libraries were constructed using the Kapa HyperPlus library preparation kit (Roche Diagnostics, Indianapolis, IN, USA). Libraries were quantified using the Kapa library quantification kit for Illumina/Bio-Rad iCycler (Roche Diagnostics) on a CFX96 real-time cycler (Bio-Rad, Hercules, CA, USA). Sequencing libraries were normalized to 2 nM, pooled, denatured, and diluted to 20 pM. The pooled samples were further diluted to a final concentration of 14 pM. Samples were sequenced on the MiSeq system (Illumina, Inc., San Diego, CA) using reagent kit v3 (600 cycles; 2 by 300 bp) (Illumina). Reads were mapped to the JE2 reference genome, and SNPs were identified using Geneious (v10).

Genomic DNA from clinical S. aureus isolates was prepared for sequencing using the Nextera XT DNA library prep kit (Illumina) and the associated protocol. Libraries were validated by running 5 μL of PCR cleanup mix on a 1% agarose gel, bead normalized, and pooled in equal volumes. Pooled normalized libraries (a 2 nM starting concentration was assumed) and PhiX were diluted and denatured according to the MiSeq system user’s guide, with a final concentration of 80 pM. The final pool was heated at 96°C for 3 min to ensure denaturation before sequencing on the MiSeq system using a read length of 2 by 300 bp, onboard fastq file generation, and sample demultiplexing, generating 0.6 million to 1.4 million paired reads per sample. Reads were processed using CLC Genomics Workbench (v.20.0.4) and the Microbial Genomics Module (v.20.1.1) “Type a Known Species” workflow. Reads were also mapped to the USA300 FPR3757 chromosome to identify single and multiple nucleotide variants relative to the laboratory strain. Multilocus sequence typing (MLST) schemes were used to identify mutations shared by a clonal complex to exclude them from further analysis.

### Statistical analysis.

All growth curve experiments were repeated three times using three technical replicates in each experiment. RT-PCR studies were performed using three independent biological replicates performed with two technical replicates in each experiment. Statistical analysis was performed using GraphPad Prism 9. Data were analyzed for normality and subsequently analyzed using one-way analysis of variance (ANOVA) with Tukey’s or Dunnett’s posttest and Student’s *t* test, as appropriate.
