# Supplementary material for: Catabolic Ornithine Carbamoyltransferase Activity Facilitates Growth of Staphylococcus aureus in Defined Medium Lacking Glucose and Arginine
Source: mBio. 2022 Apr 27;13(3):e00395-22. doi: 10.1128/mbio.00395-22 (PMC9239276; doi:10.1128/mbio.00395-22)
Supplement: FIG S1 [file mbio.00395-22-s0001.pdf]

Figure S1

| | 1 … 60 |
|---|---|
| JE2 | MPKKSVRHIKIREIISNEQIETQDELVKRLNDYDLNVTQATVSRDIKELQLIKVPIPSGQ |
| JE2 *ahrC*$_{C124F}$ | MPKKSVRHIKIREIISNEQIETQDELVKRLNDYDLNVTQATVSRDIKELQLIKVPIPSGQ |
| JE2 *ahrC*$_{K4N}$ | MPKNSVRHIKIREIISNEQIETQDELVKRLNDYDLNVTQATVSRDIKELQLIKVPIPSGQ |

| | 61 … 118 |
|---|---|
| JE2 | YVYSLPNDRKFHPLEKLGRYLMDSFVNIDGTDNLLVLKTLPGNAQSIGAILDQINWEE |
| JE2 *ahrC*$_{C124F}$ | YVYSLPNDRKFHPLEKLGRYLMDSFVNIDGTDNLLVLKTLPGNAQSIGAILDQINWEE |
| JE2 *ahrC*$_{K4N}$ | YVYSLPNDRKFHPLEKLGRYLMDSFVNIDGTDNLLVLKTLPGNAQSIGAILDQINWEE |

| | 119 … 150 |
|---|---|
| JE2 | VLGTICGDDTCLIICRSKEASDEIKSRIFNLL |
| JE2 *ahrC*$_{C124F}$ | VLGTIFGDDTCLIICRSKEASDEIKSRIFNLL |
| JE2 *ahrC*$_{K4N}$ | VLGTICGDDTCLIICRSKEASDEIKSRIFNLL |
