# Supplementary material for: Catabolic Ornithine Carbamoyltransferase Activity Facilitates Growth of Staphylococcus aureus in Defined Medium Lacking Glucose and Arginine
Source: mBio. 2022 Apr 27;13(3):e00395-22. doi: 10.1128/mbio.00395-22 (PMC9239276; doi:10.1128/mbio.00395-22)
Supplement: TABLE S1 [file mbio.00395-22-s0008.docx]

**Table S1. Identified mutations in JE2 isolates able to grow in CDM-R**

| **Isolate** | **Nucleotide^1^** | **Nucleotide Change** | **Amino Acid Change** | **Protein Effect** | **Product** |
| --- | --- | --- | --- | --- | --- |
| 8 (P*arc*3*)* | 2784204  2784202 | T🡪A  -T |  |  | *arcA1B1D1C1* upstream region *arcA1B1D1C1* upstream region |
| 17 (C124F) | 1639590 | C🡪A | C🡪F | Substitution | AhrC repressor |
| 18 (P*arc*2) | 804659  1564471  2711153  2784204 | A🡪T  C🡪T  T🡪G  T🡪A | L🡪I  G🡪D  I🡪L | Substitution  Substitution  Substitution | Di/Tripeptide permease YjdL  Phase lysin  Acetyltransferase  *arcA1B1D1C1* upstream region |
| 21 (K4N) | 1550253  1561036  1639949 | C🡪A  T🡪A  T🡪A | P🡪H  K🡪N | Substitution  Substitution | L-asparaginase  Intergenic region  AhrC repressor |
| 22 (P*arc*1) | 304912  505342  1034607  1721293  2784215 | G🡪A  CGG🡪TTT  G🡪T  C🡪A  T🡪A | A🡪T  GG🡪GL  V🡪L | Substitution  Substitution  Substitution | Glycosyl transferase  PTA System, trehalose  Intergenic region  Peptidase, U32 family  *arcA1B1D1C1* upstream region |

^1^Compared to the *S. aureus* JE2 reference genome NCBI Reference Sequence: NZ_CP020619.1
