# Supplementary figures and images for: Catabolic Ornithine Carbamoyltransferase Activity Facilitates Growth of Staphylococcus aureus in Defined Medium Lacking Glucose and Arginine
Source: mBio. 2022 Apr 27;13(3):e00395-22. doi: 10.1128/mbio.00395-22 (PMC9239276; doi:10.1128/mbio.00395-22)

Figure S2

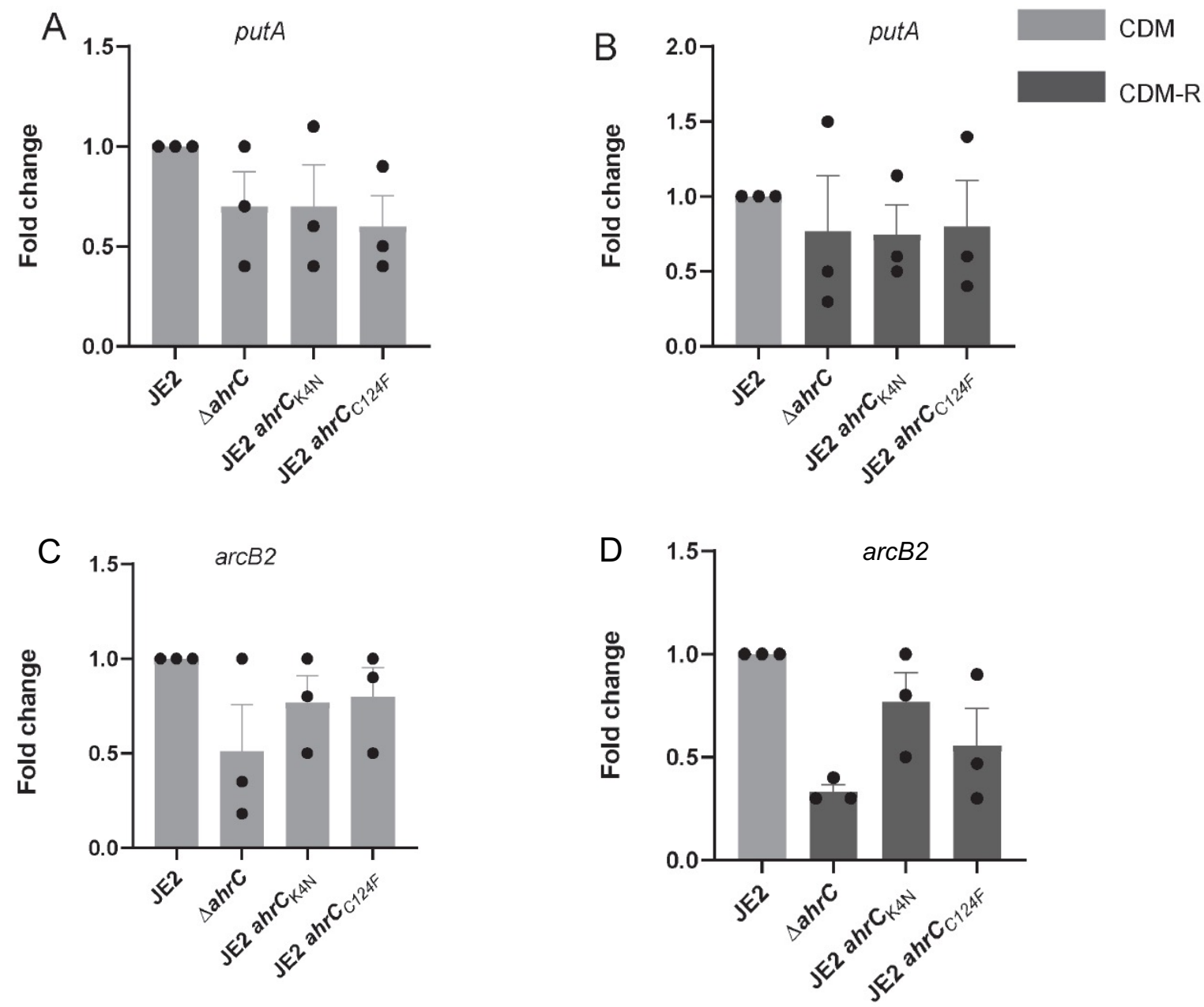

Supplement: FIG S2 [file mbio.00395-22-s0002.pdf]

Figure S3

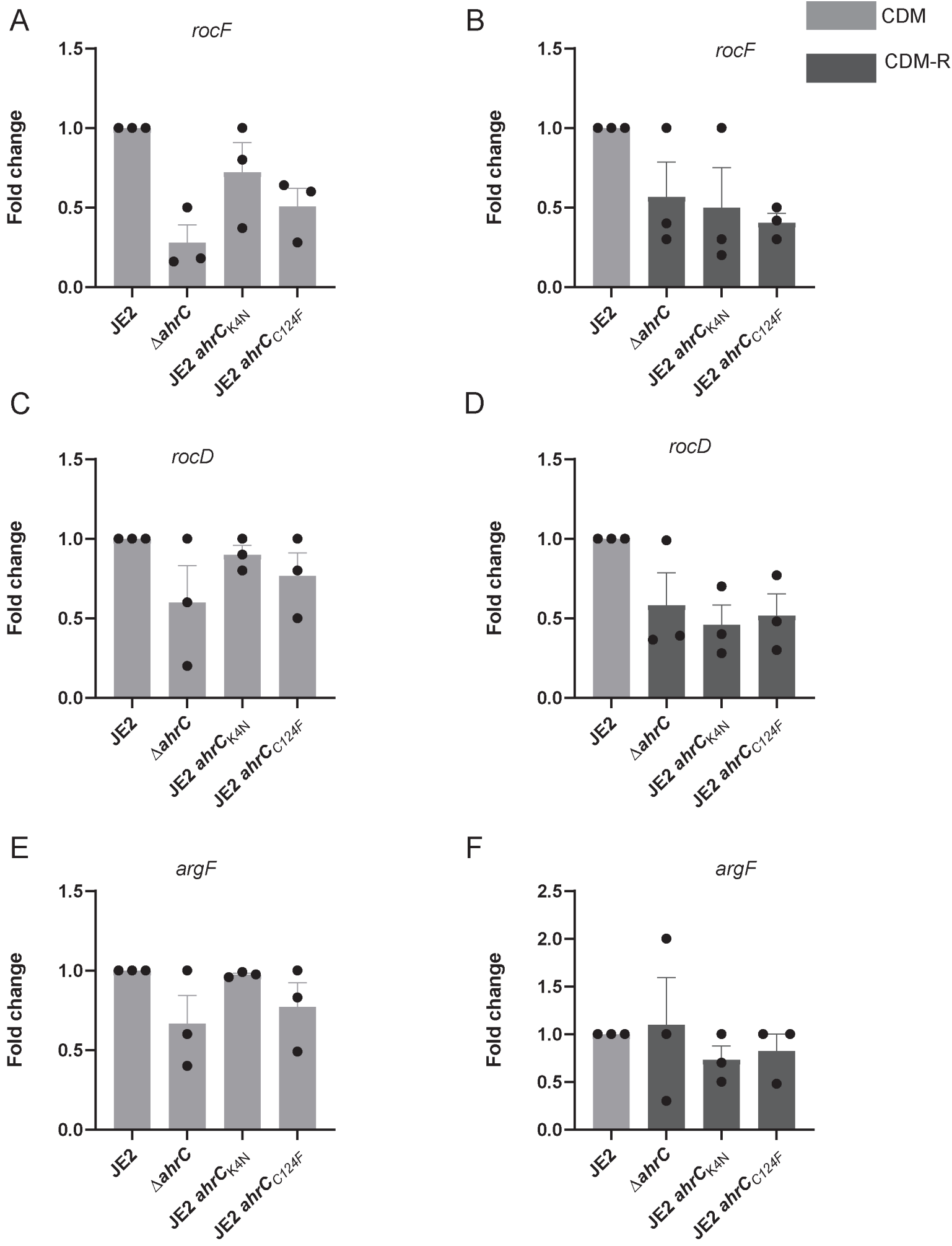

Supplement: FIG S3 [file mbio.00395-22-s0003.pdf]

Figure S4

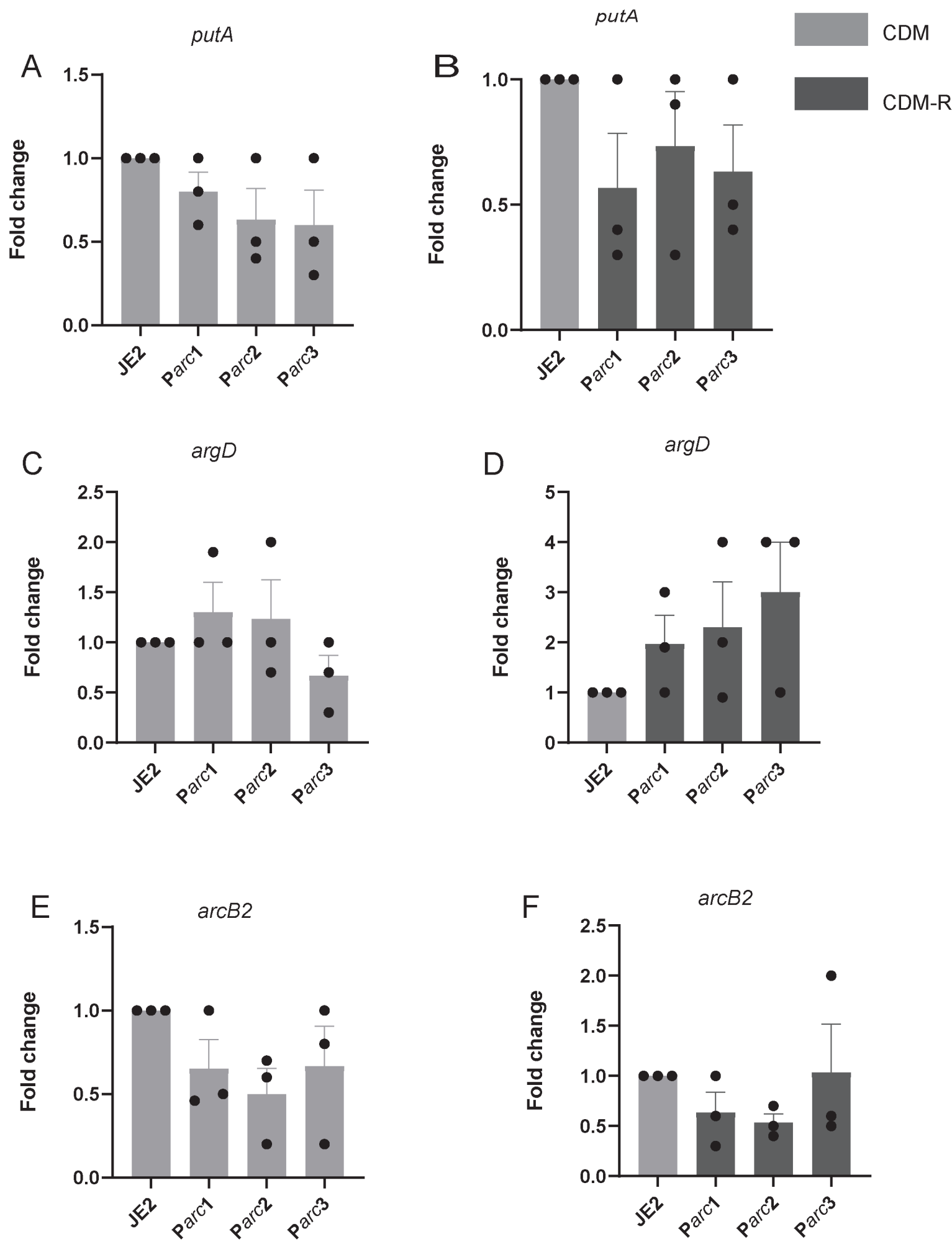

Supplement: FIG S4 [file mbio.00395-22-s0004.pdf]

Figure S5

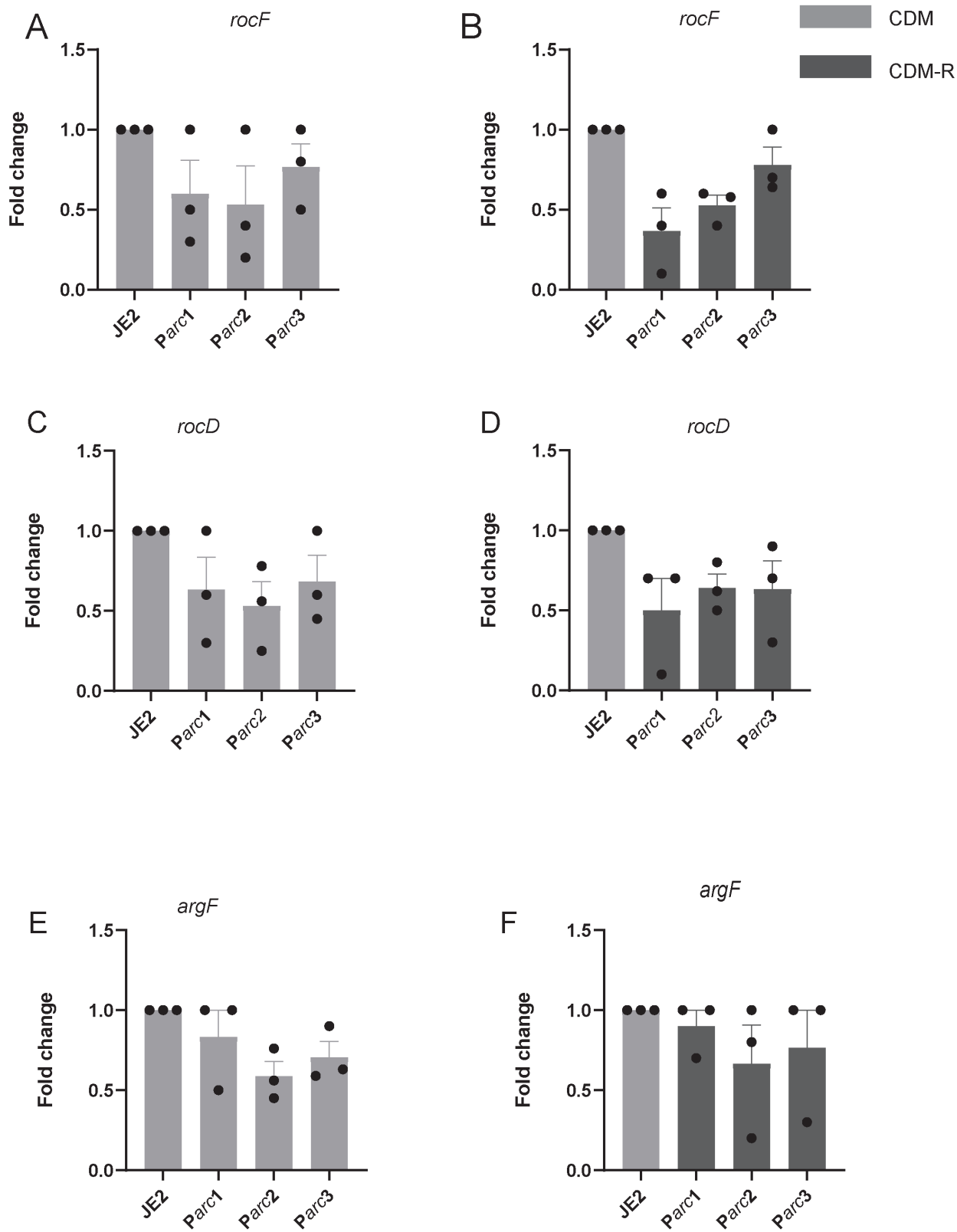

Supplement: FIG S5 [file mbio.00395-22-s0005.pdf]
