# Supplementary material for: Catabolic Ornithine Carbamoyltransferase Activity Facilitates Growth of Staphylococcus aureus in Defined Medium Lacking Glucose and Arginine
Source: mBio. 2022 Apr 27;13(3):e00395-22. doi: 10.1128/mbio.00395-22 (PMC9239276; doi:10.1128/mbio.00395-22)

Growth in CDMG-R

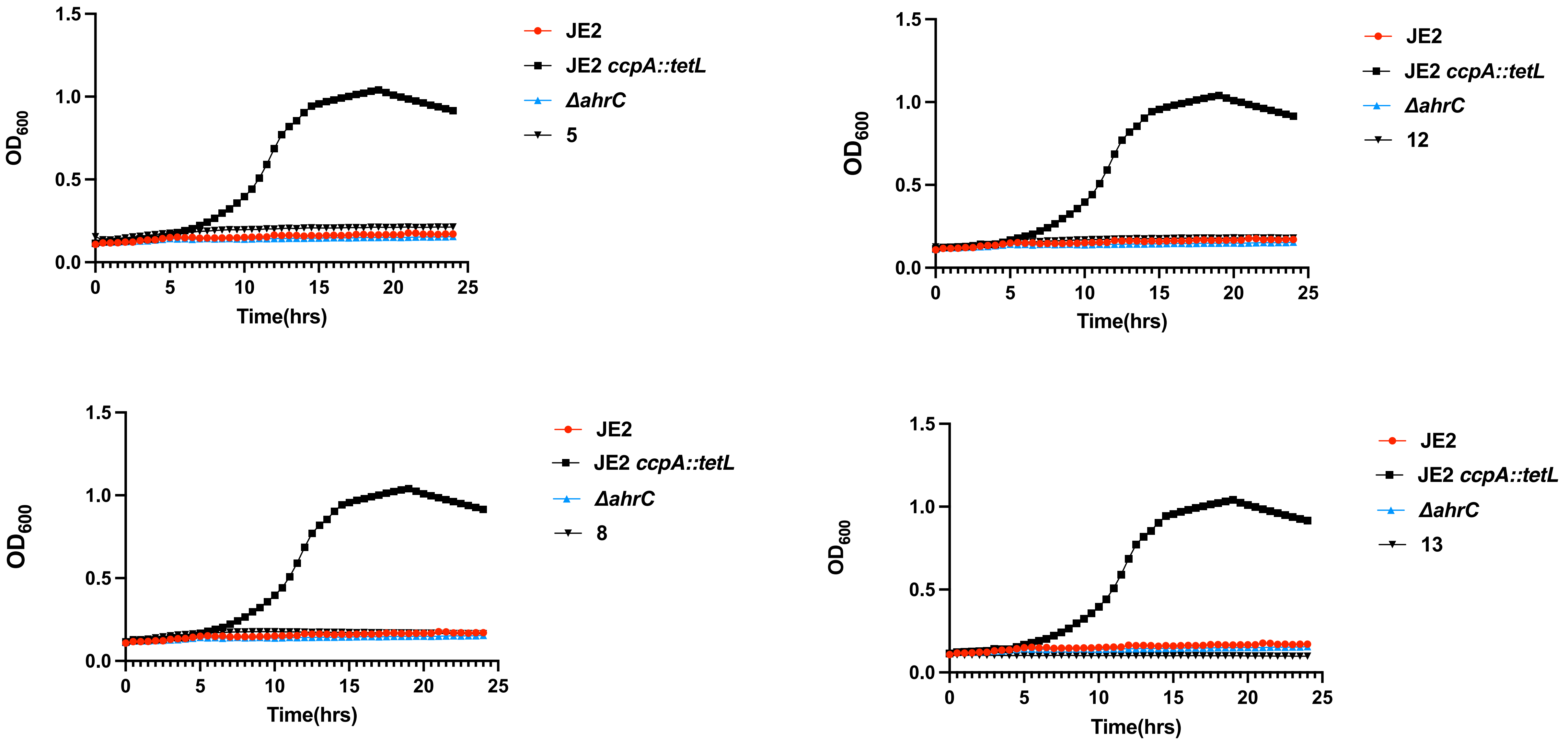

Growth in CDMG-R

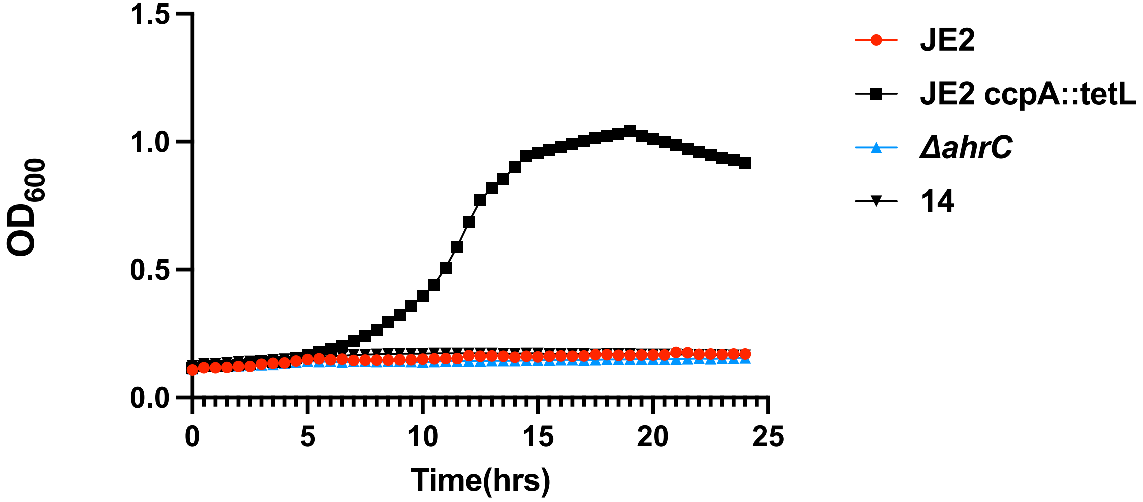

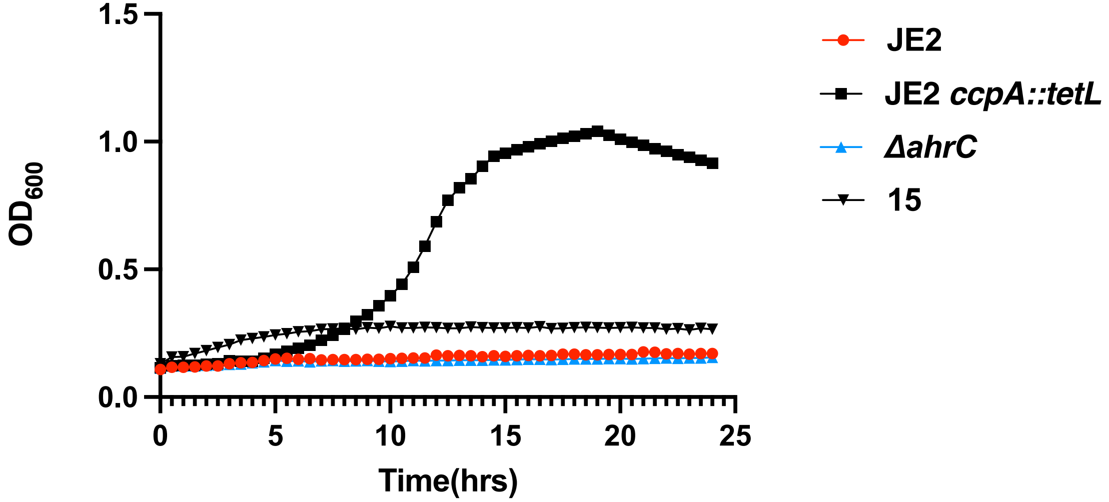

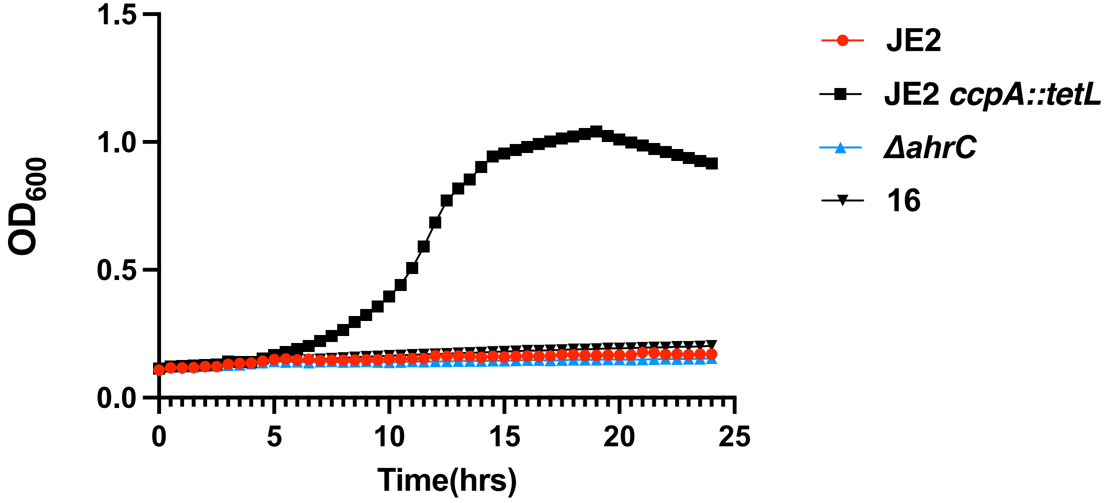

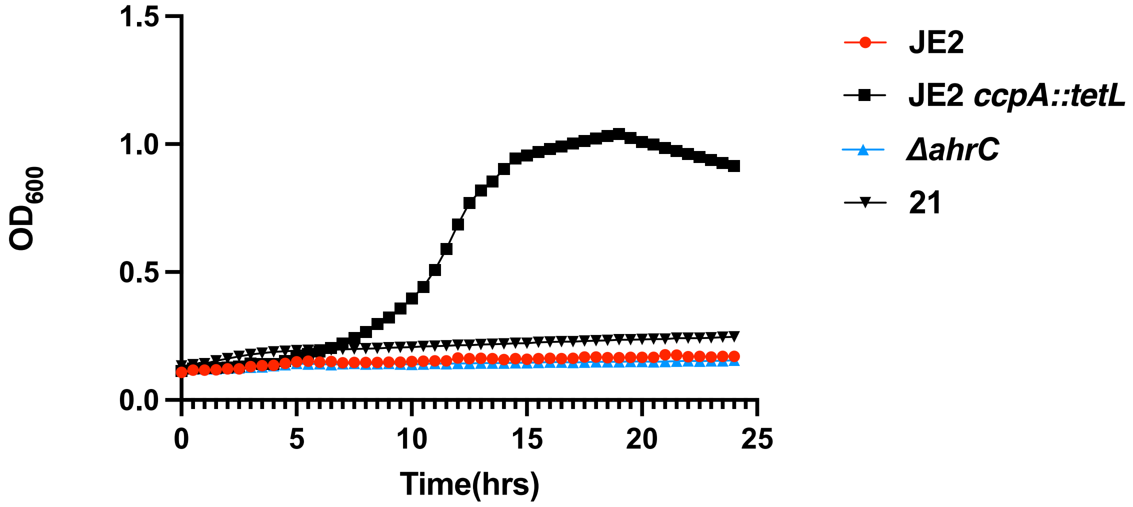

Growth in CDMG-R

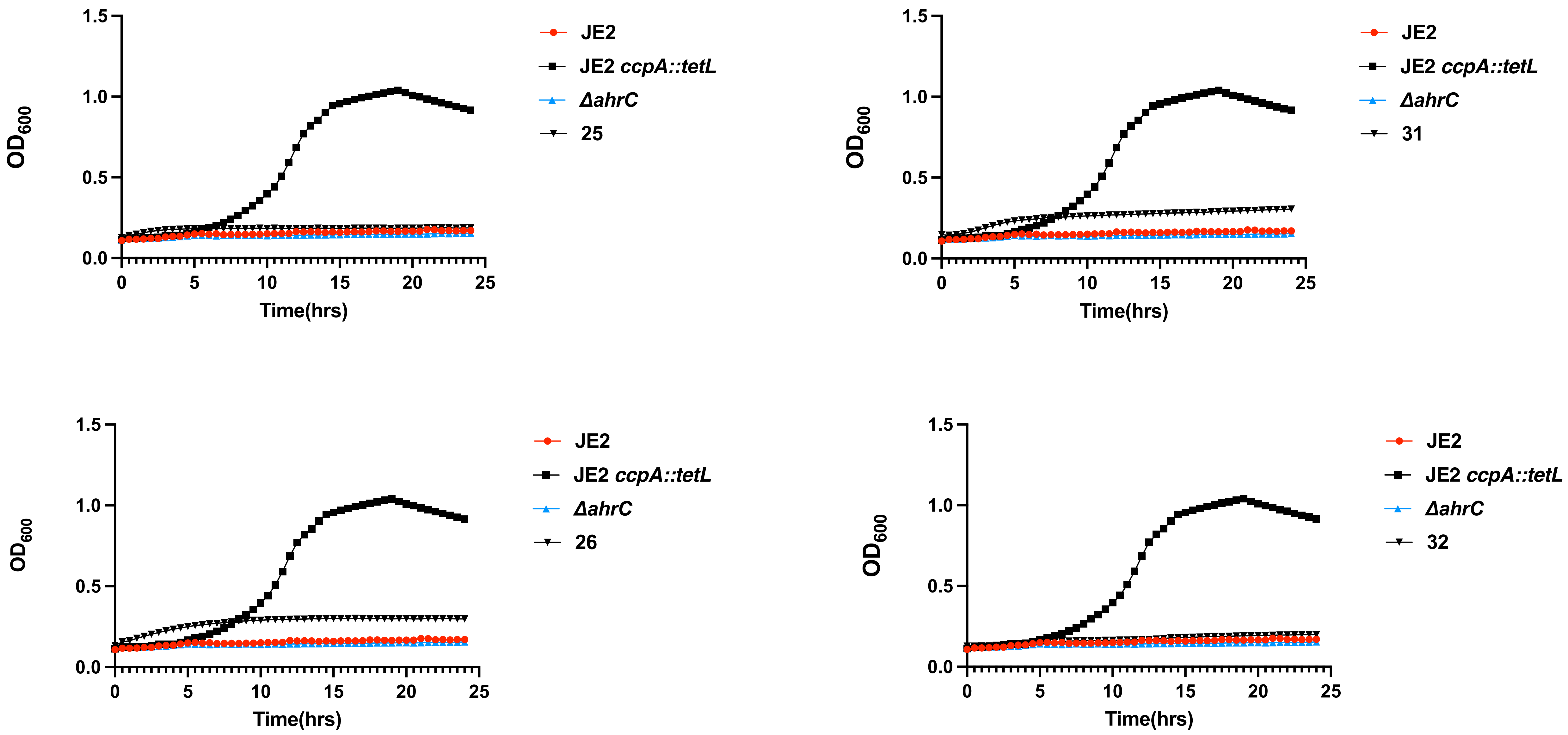

Growth in CDMG-R

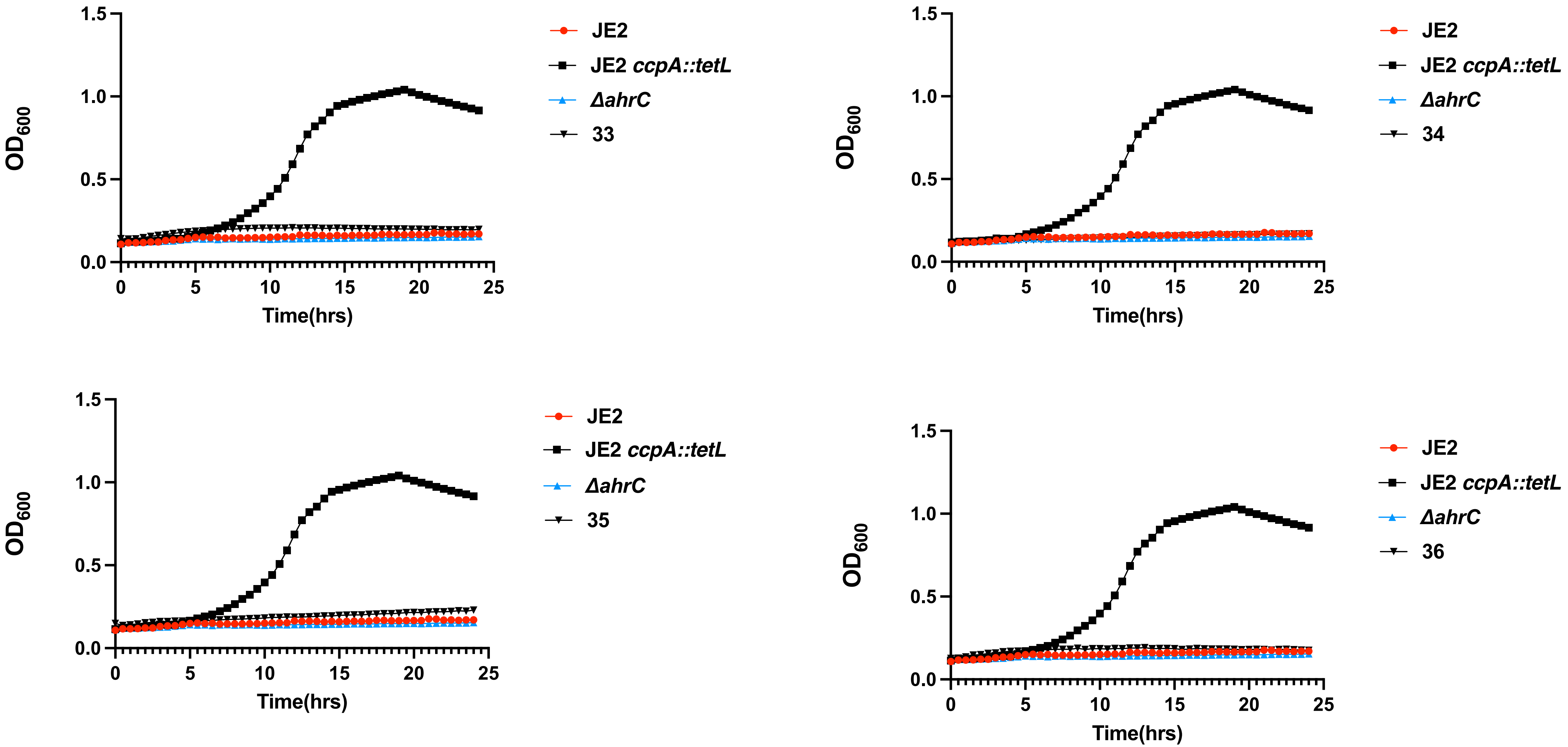

Growth in CDMG-R

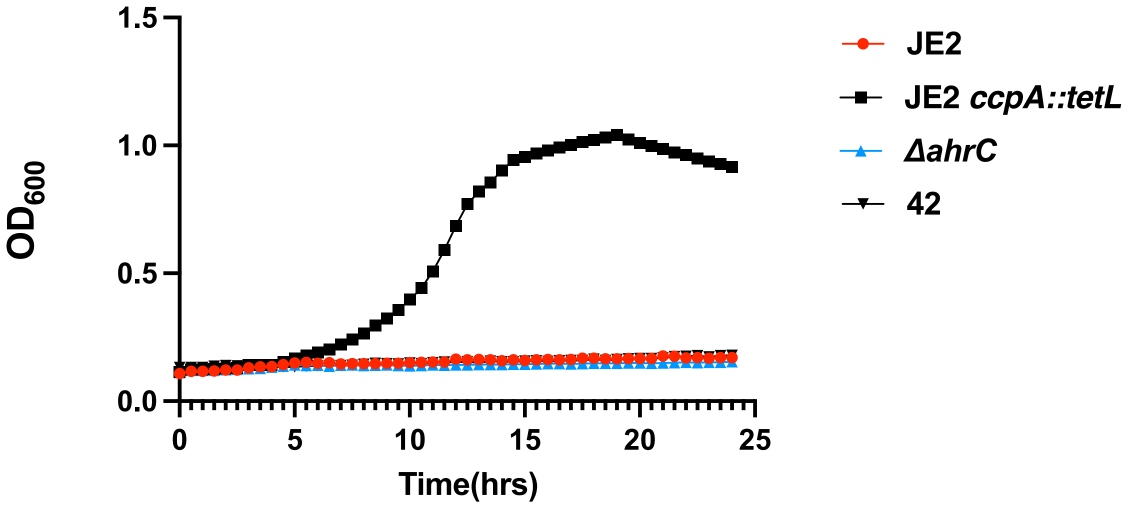

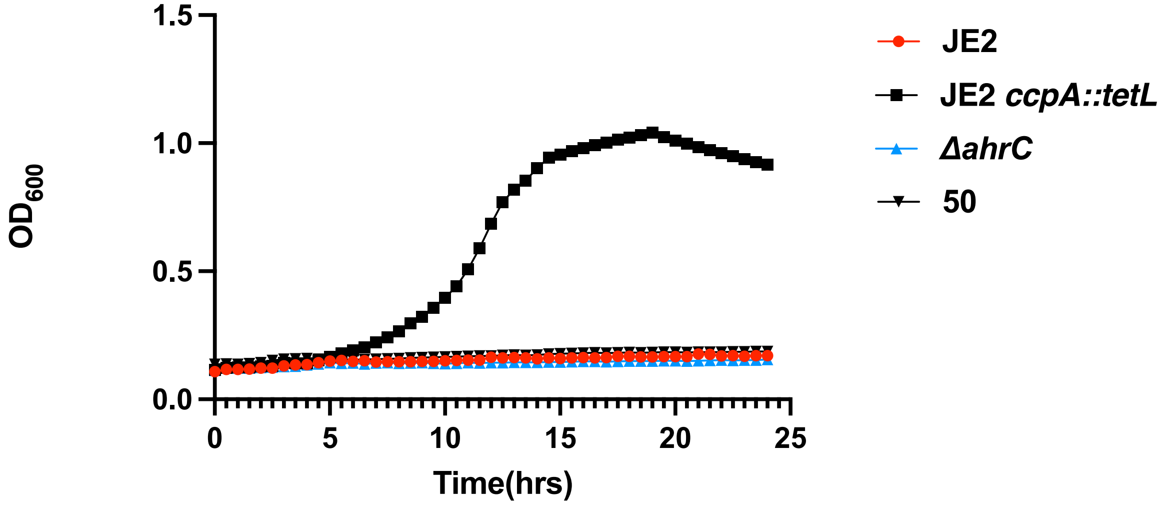

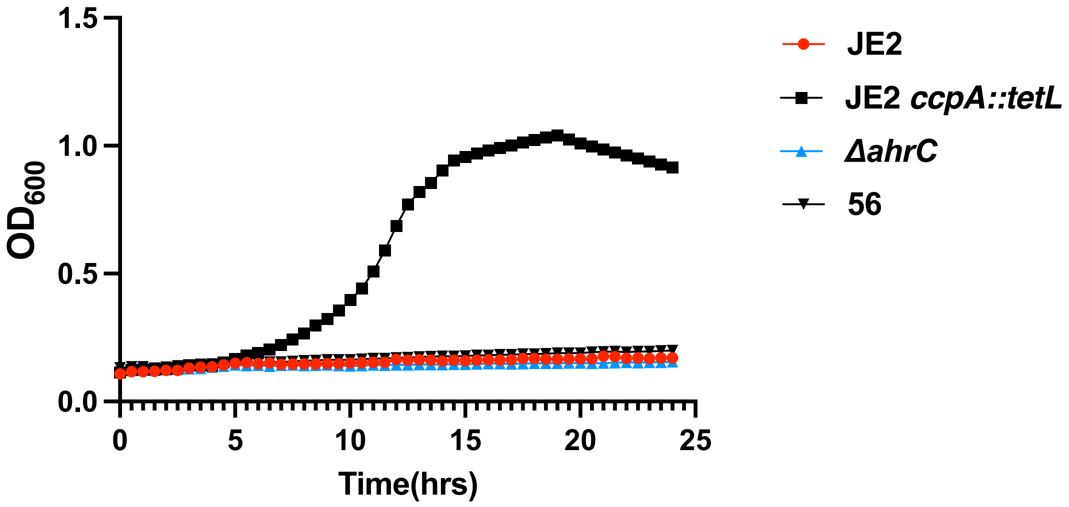

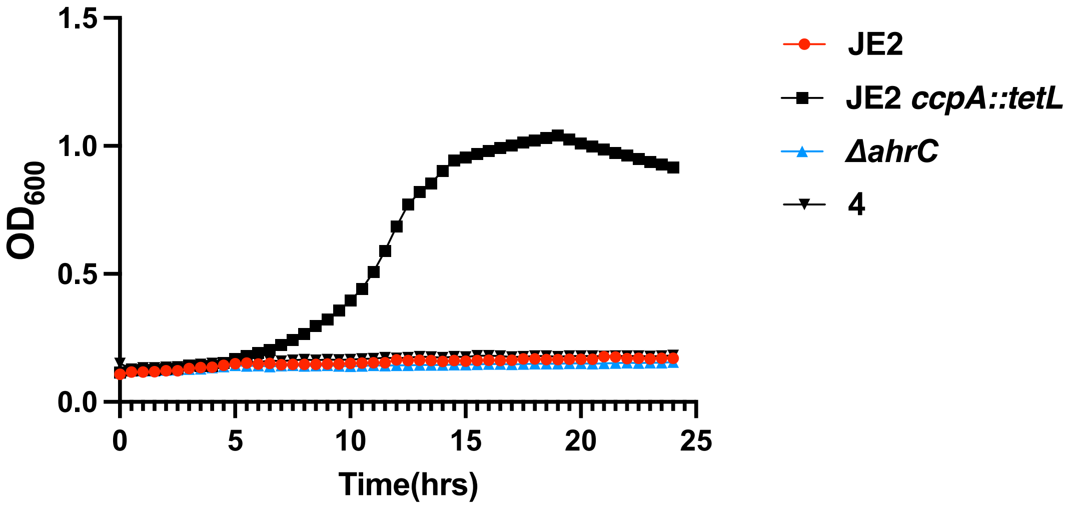

Growth in CDMG-R

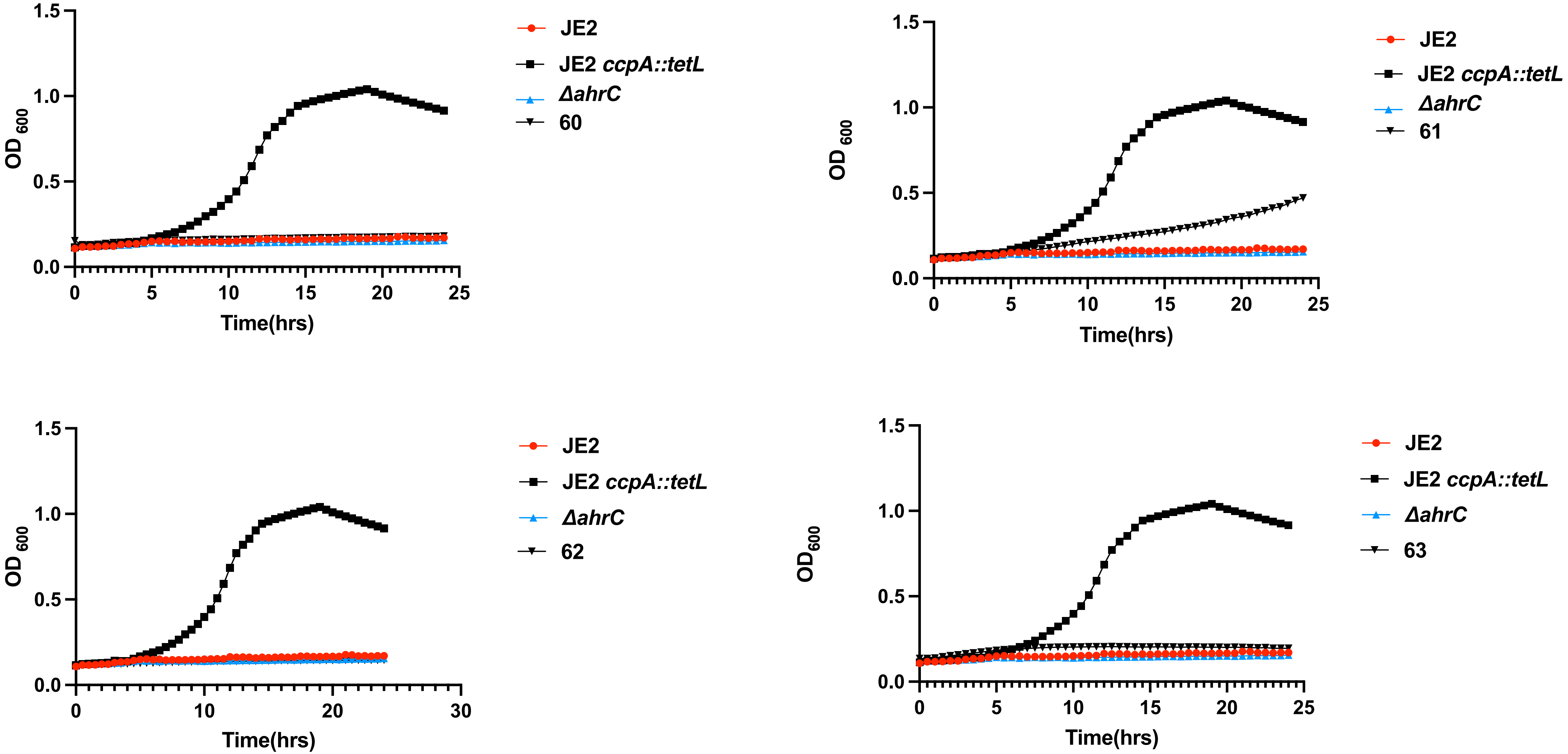

Growth in CDMG-R

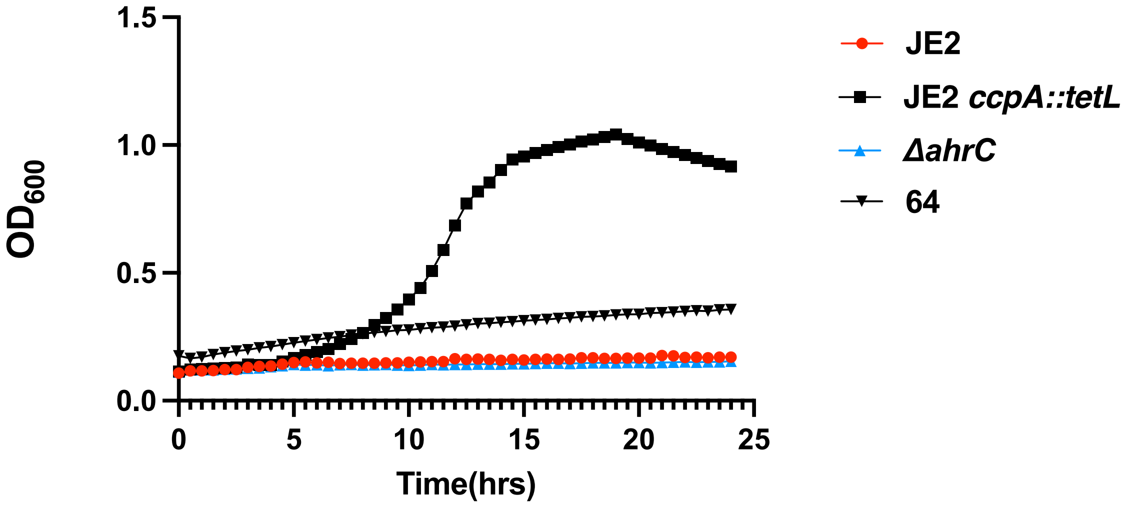

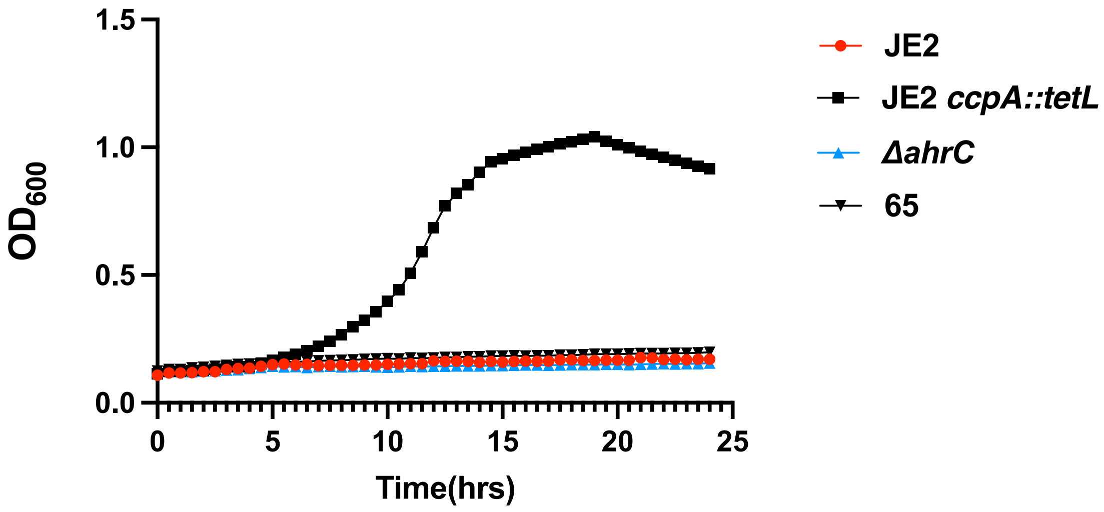

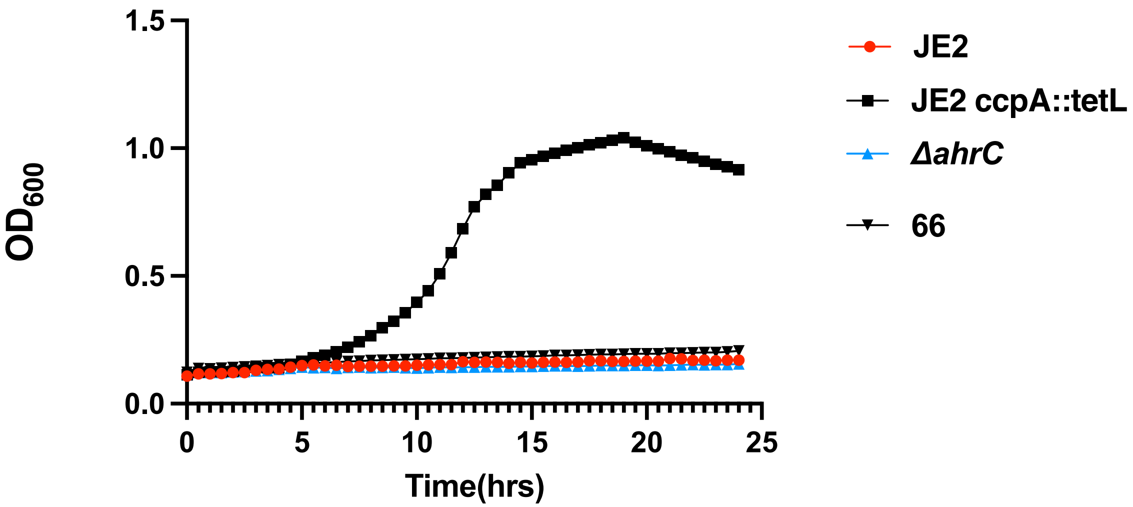

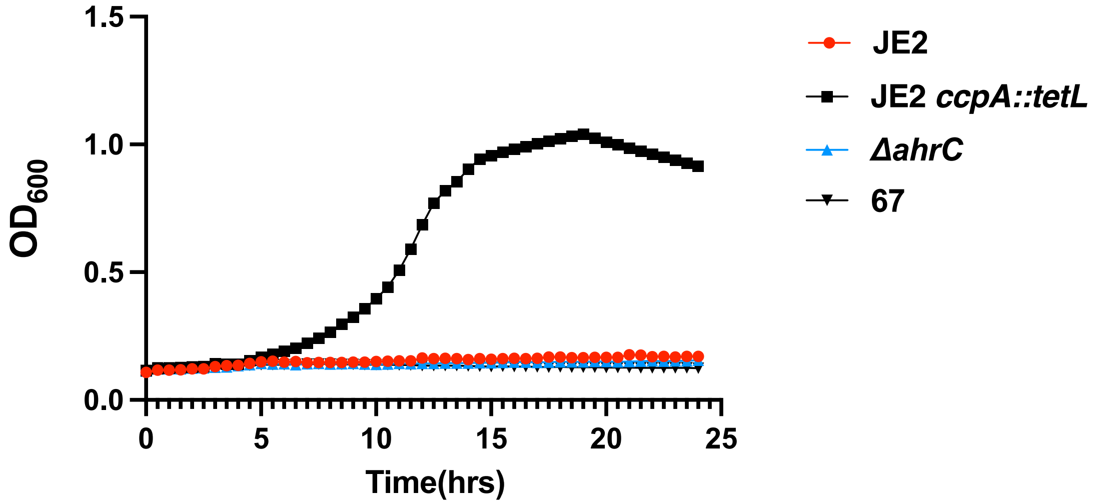

Growth in CDMG-R

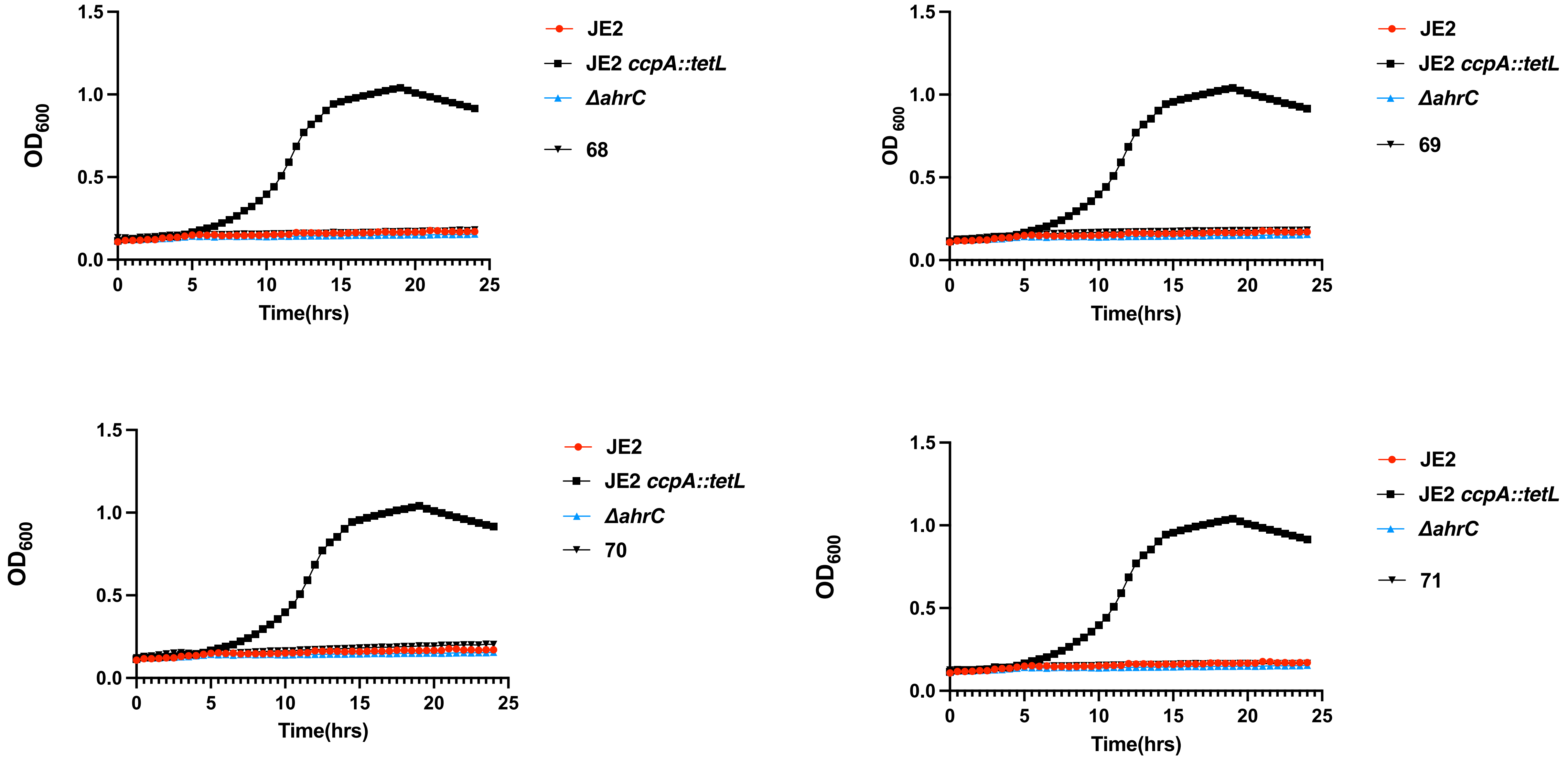

Growth in CDMG-R

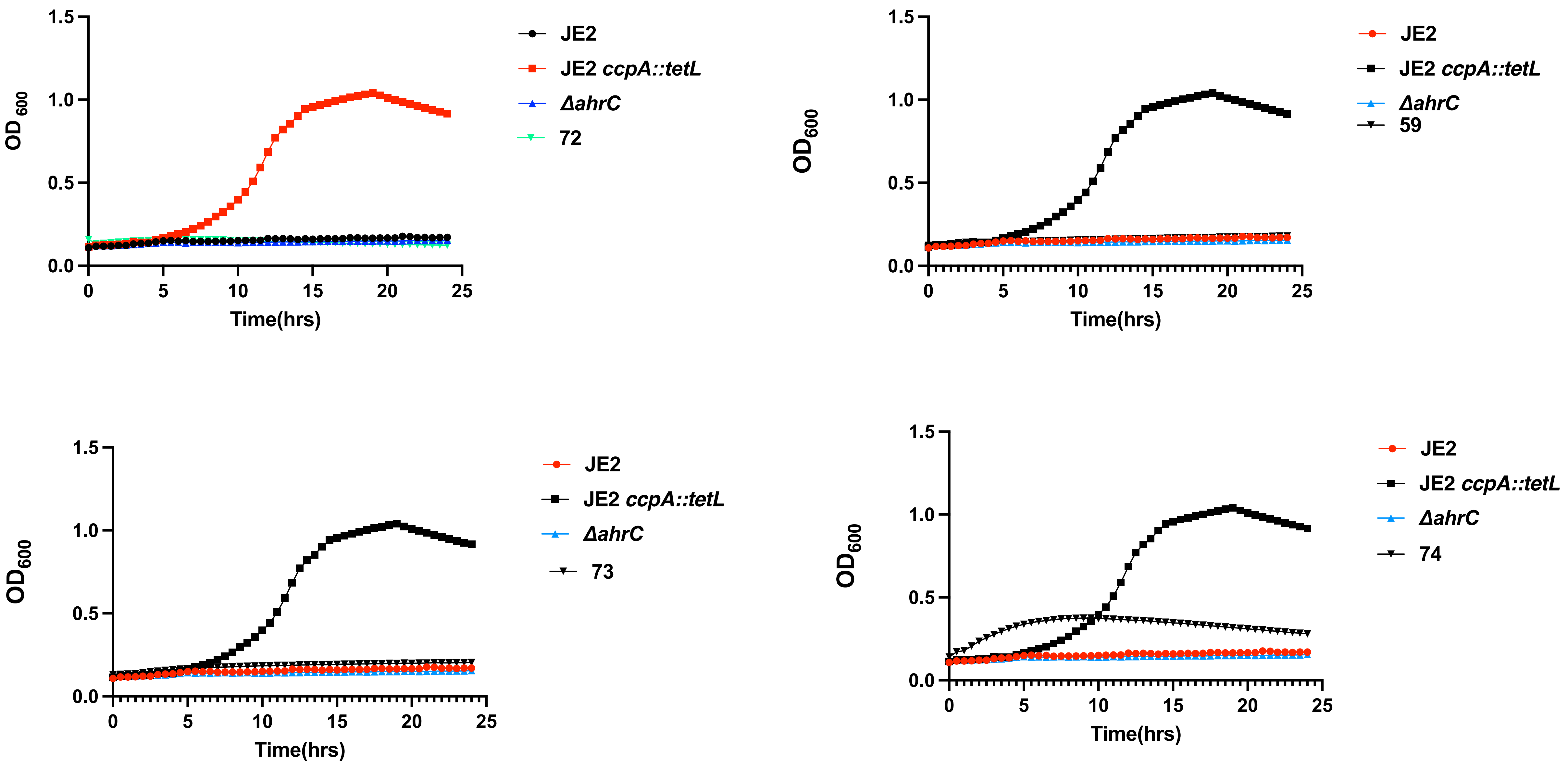

Growth in CDMG-R

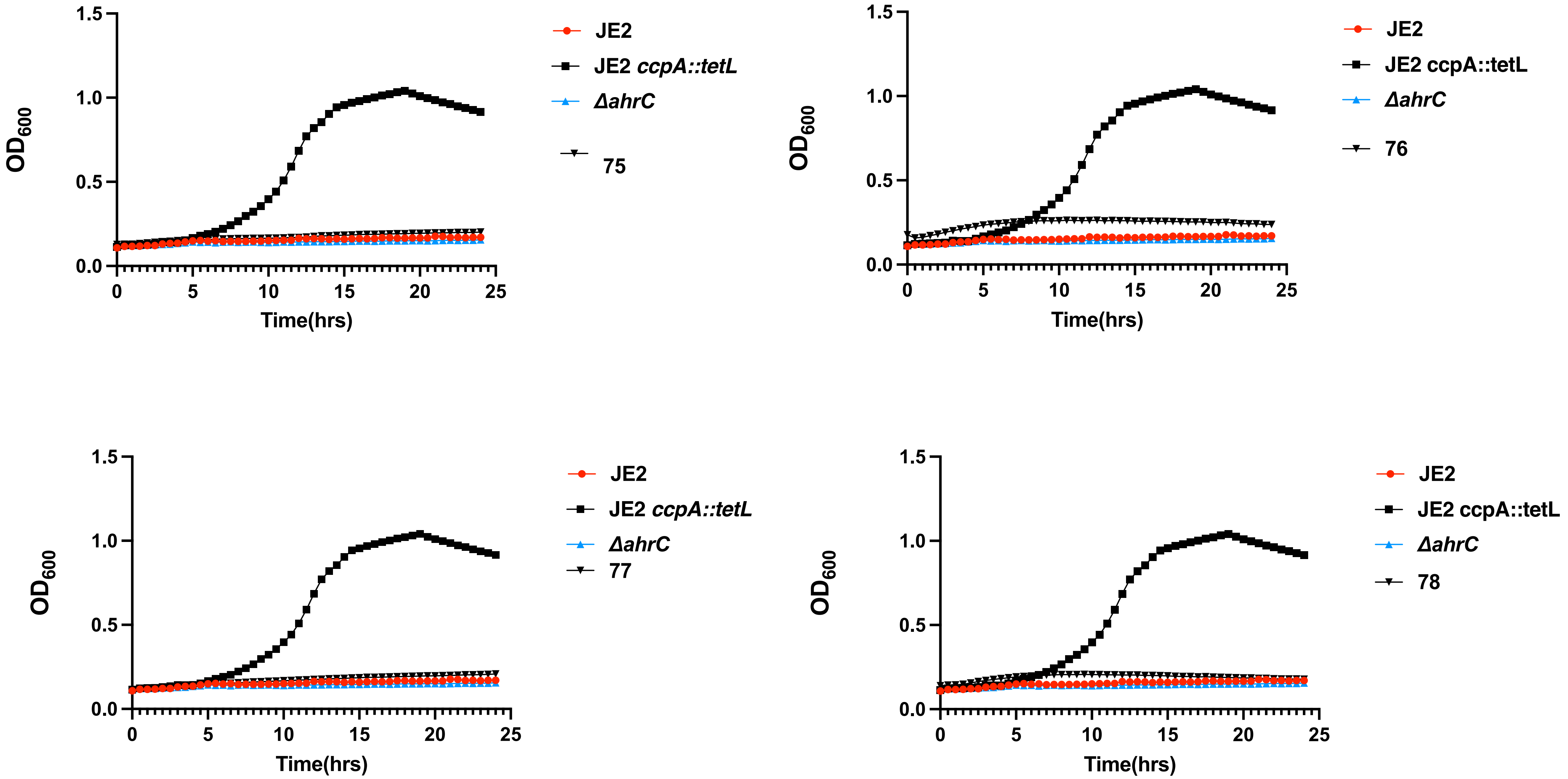

Growth in CDMG-R

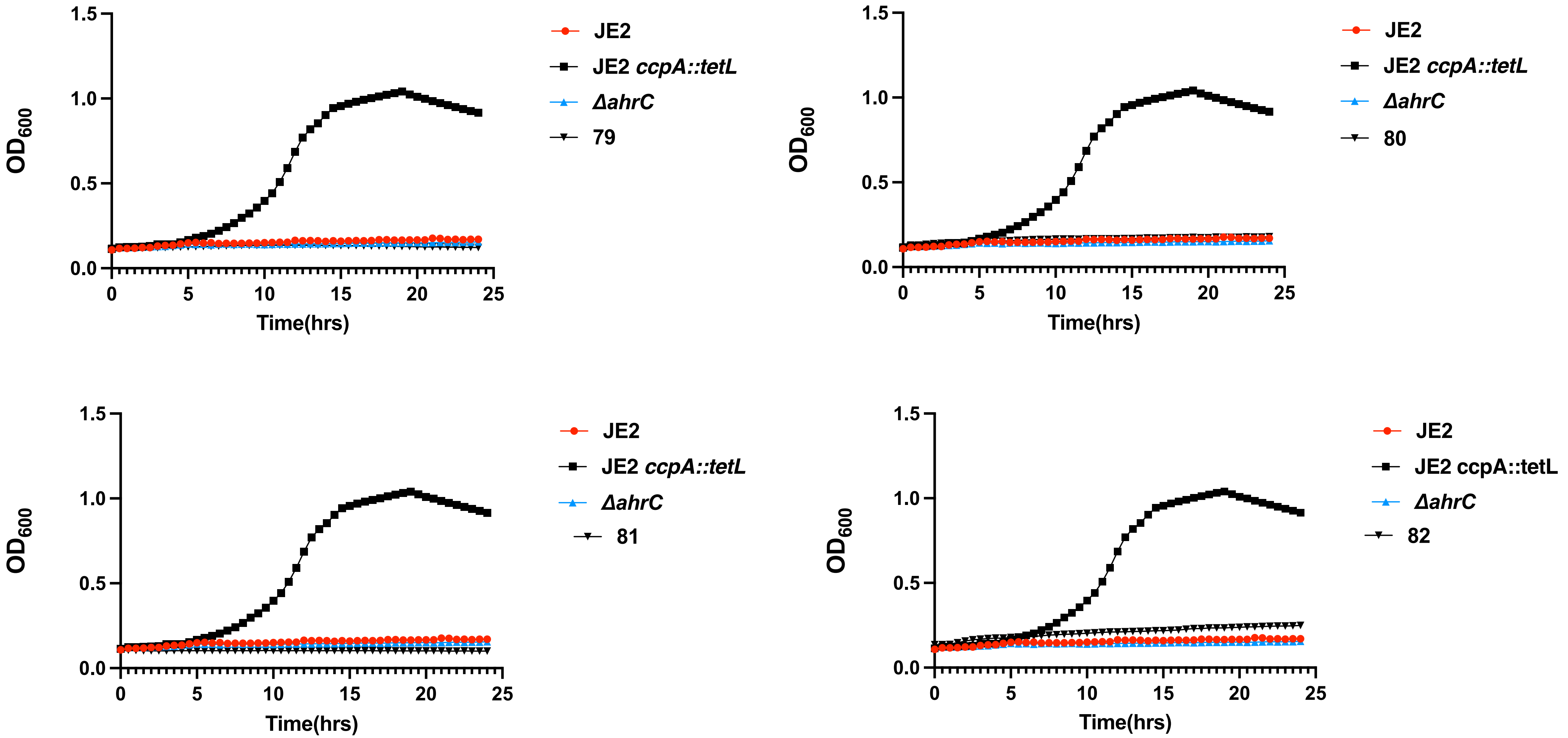

Supplement: FIG S6 [file mbio.00395-22-s0006.pdf]
