# Supplementary material for: Catabolic Ornithine Carbamoyltransferase Activity Facilitates Growth of Staphylococcus aureus in Defined Medium Lacking Glucose and Arginine
Source: mBio. 2022 Apr 27;13(3):e00395-22. doi: 10.1128/mbio.00395-22 (PMC9239276; doi:10.1128/mbio.00395-22)
Supplement: TABLE S2 [file mbio.00395-22-s0009.docx]

**Table S2. Strains, primers and probes used in study**

**Strains**

| **Bacterial strain/Plasmids** | **Relevant phenotype** | **Source** |
| --- | --- | --- |
| pUC19 | Gram-negative origin of replication, Amp^R^ | Invitrogen |
| pROJ6448 | pE194 containing pC221 nick site functioning in conjugative mobilization, temp sensitive gram-positive origin of replication, Erm | [1] |
| pNF293 | *ahrC* allelic replacement vector for makerless, in-frame *ahrC* mutant; Amp^R^, Erm^R^ | This study |
| *S. aureus* RN4220 | Restriction deficient NCTC8325-4 | [2] |
| *S. aureus* JE2 | LAC cured of all 3 native plasmids | [3] |
| *S. aureus* JE2 *putA::*φΝΣ | *bursa aurealis* insertion in proline dehydrogenase backcrossed into JE2 using Φ11 | [4] |
| *S. aureus* JE2 *argC::*φΝΣ | *bursa aurealis* insertion in N-acetyl-gamma-glutamyl-phosphate reductase backcrossed into JE2 using Φ11 | [4] |
| *S. aureus* JE2 *rocA::* φΝΣ | *bursa aurealis* insertion in pyrroline-5-carboxylate dehydrogenase  backcrossed into JE2 using Φ11 | [4] |
| *S. aureus* JE2 *proC::* φΝΣ | *bursa aurealis* insertion in pyrroline-5-carboxylate reductase  backcrossed into JE2 using Φ11 | [4] |
| *S. aureus* JE2 *argR2::* φΝΣ (*spec*) | *bursa aurealis* insertion in *argR2*  backcrossed into JE2 using Φ11. Erythromycin resistance cassette replaced by a spectinomycin resistance cassette via allelic replacement | [3, 5] |
| *S. aureus* JE2 *argR1::* φΝΣ | *bursa aurealis* insertion in *argR1*  backcrossed into JE2 using Φ11 | [3] |
| *S. aureus* JE2 *argR1::* φΝΣ *argR2::* φΝΣ (*spec*) | *argR2::* φΝΣ (*spec*) marker transduced via Φ11 to JE2 *argR1::* φΝΣ | This study |
| pBK123 | Chloramphenicol-resistant derivative of shuttle plasmid pCN51 | [6, 7] |
| pNF378 | pBK123 with the *arcA1B1D1C1* operon ORF cloned behind the cadmium-inducible promoter at the *Sal*I/*Xma*I sites. | This study |
| pNF379 | pBK123 with the *argGH* operon ORF cloned behind the cadmium-inducible promoter at the *Sal*I/*Xma*I sites. | This study |
| pNF406 | pBK123 with the *ahrC* ORF cloned behind the cadmium-inducible promoter at the *Sal*I/*Xma*I sites. | This study |
| pNF407 | pBK123 with the *arcB1* ORF cloned behind the cadmium-inducible promoter at the *Sal*I/*Xma*I sites. | This study |
| *S. aureus* JE2 *ΔahrC* | Markerless deletion in SAUS300_1469, arginine repressor | This study |

**Primers**

| **Name** | **Sequence** | **Comments** |
| --- | --- | --- |
| 1 | CCTTATGGCGATGATTGG TTTG | Forward primer for *S. aureus* JE2 *putA* transcriptional analysis by RT-PCR |
| 2 | CCAGCAGGTTTCACAAAT TCTT | Reverse primer for *S. aureus* JE2 *putA* transcriptional analysis by RT-PCR |
| 3 | GGTCTTGGTAGAACTGGG AAAT | Forward primer for *S. aureus* JE2 *argD* transcriptional analysis by RT-PCR |
| 4 | CAAGTACAGCAGATACAG GGTATAA | Reverse primer for *S. aureus* JE2 *argD* transcriptional analysis by RT-PCR |
| 5 | CTGTAGAGGATACTGCGA AAGT | Forward primer for *S. aureus* JE2 *arcB1* transcriptional analysis by RT-PCR |
| 6 | GGATGCCAATCGTCTGTT AATC | Reverse primer for *S. aureus* JE2 *arcB1* transcriptional analysis by RT-PCR |
| 7 | CATTTGAAACAGCGGCTT ATGA | Forward primer for *S. aureus* JE2 *arcB2* transcriptional analysis by RT-PCR |
| 8 | ACACGAGCAGTATCTTTG GTAG | Reverse primer for *S. aureus* JE2 *arcB2* transcriptional analysis by RT-PCR |
| 9 | AAGAACGCGATGTGCATT TG | Forward primer for *S. aureus* JE2 *argF* transcriptional analysis by RT-PCR |
| 10 | AGCACACGTGCAGTATCT TTA | Reverse primer for *S. aureus* JE2 *argF* transcriptional analysis by RT-PCR |
| 11 | CAGAGCGAATGAATGTGG TATTT | Forward primer for *S. aureus* JE2 *argGH* transcriptional analysis by RT-PCR |
| 12 | TCAGCAGTATCTGGTGTT TCTT | Reverse primer for *S. aureus* JE2 *argGH* transcriptional analysis by RT-PCR |
| 13 | CGGTAGGTTCAGTATCAG CAATAA | Forward primer for *S. aureus* JE2 *rocF* transcriptional analysis by RT-PCR |
| 14 | TTTCCACTTGGTGACTCT TCAG | Reverse primer for *S. aureus* JE2 *rocF* transcriptional analysis by RT-PCR |
| 15 | ATGTCTTTACACCTGGCT CAC | Forward primer for *S. aureus* JE2 *rocD* transcriptional analysis by RT-PCR |
| 16 | GGCCTGGTAAATCCTCAT CAA | Reverse primer for *S. aureus* JE2 *rocD* transcriptional analysis by RT-PCR |
| 17 | CAAATGATCACAGCATTT GGTACAG | Forward primer for *S. aureus* JE2 *gyrB* transcriptional analysis by RT-PCR. |
| 18 | CGGCATCAGTCATAATGA CGAT | Reverse primer for *S. aureus* JE2 *gyrB* transcriptional analysis by RT-PCR. |
| 19 | GAAGAGCGTGAAAGTGGT AAAG | Forward primer for detection of *tpiA* transcript by qPCR |
| 20 | GATTGCCCAGATTGGTTC ATAAG | Reverse primer for detection of *tpiA* transcript by qPCR |
| 104 | CTGGTAATGCACAATCTA TTG GAG | Forward primer for S. aureus JE2 ahrC transcriptional analysis by RT-PCR |
| 105 | GCTTCTTTG CTTCGACAA ATAA | Reverse primer for S. aureus JE2 ahrC transcriptional analysis by RT-PCR |
| 124 | gaaggtcaatgtctgaacctgcag gtcgacAAGGAGGAAATA GACATGAC | Forward primer for cloning arcA1B1DC behind the cadmium-inducible promoter of pBK123. |
| 125 | gcgcgcctgaattcgagctcggtaccc gggCTACATGTGAATGTGTGTAC | Reverse primer for cloning arcA1B1DC behind the cadmium-inducible promoter of pBK123. |
| 126 | gaaggtcaatgtctgaacctgcaggtc gACTACAAGAGGTGAATTTT ATG | Forward primer for cloning argGH behind the cadmium-inducible promoter of pBK123. |
| 127 | gcgcgcctgaattcgagctcggtaccc gggTTATTGTGATAGTAATTG TTTAGC | Reverse primer for cloning argGH behind the cadmium-inducible promoter of pBK123. |
| 110 | AGAAAAGAAGGAAAACTAGC | pBK123 sequencing primer - flanks MCS |
| 111 | CAAAATTA ACATGTCAACG | pBK123 sequencing primer - flanks MCS |
| 172 | gaaggtcaatgtctgaacctgcaggtc gacGGCCCTAGATGTATGAGT CAACC | Forward primer for cloning arcB1 behind the cadmium-inducible promoter of pBK123. |
| 173 | gcgcgccctgaattcgagctcggtacc cggggTTAACTCCCCAATGT TGCTGC | Reverse primer for cloning arcB1 behind the cadmium-inducible promoter of pBK123. |
| 174 | gaaggtcaatgtctgaacctgcaggtc gacCGTAATCAGAGGTGTTTA CTGTGC | Forward primer for cloning ahrC behind the cadmium-inducible promoter of pBK123. |
| 175 | gcgcgccctgaattcgagctcggtacc cggggTGTAACATAAACTCA TCGCATCC | Reverse primer for cloning ahrC behind the cadmium-inducible promoter of pBK123. |
| 1999 | CCCGGGGAATTCTCGGCG GTCAAATGTTAGAT | 5’ Forward ahrC contains EcoRI* restriction site |
| 2000 | CCCGGGGGATCCCACAGT AAACACCTCTGATTACGAA | 5’ Reverse ahrC contains BamHI* restriction site |
| 2211 | CCCGGGGGATCCTAAGGA TGCGATGAGTTTATG | 3’ Forward ahrC contains BamHI* restriction site |
| 2212 | CCCGGGCTGCAGCGATCA GTGATAGCATTTTC | 3’ Reverse ahrC contains PstI* restriction site |
| 2944 | TCTTCAAACAATTGACGCTTA TT | Forward ahrC mutant confirmation |
| 2945 | CCTTCGATGACAGCCTTTT | Reverse ahrC mutant confirmation |

*Restriction site denoted in italics

**Probes**

| Name | Sequence | 5' modification | 3' modification | Comments |
| --- | --- | --- | --- | --- |
| Probe 1 | TGAGAAGAT TAGCAGAAC GCCCACA | 6-FAM | BHQ-1 | Taqman probe for detection of *putA* transcript by qPCR |
| Probe 2 | ATGGAATGG GAGCAAGTC GTTCCA | HEX | BHQ-1 | Taqman probe for detection of *argD* transcript by qPCR |
| Probe 3 | CACCGGTACACCAGAGAA CTT CGC | 6-FAM | BHQ-1 | Taqman probe for detection of *arcB1* transcript by qPCR |
| Probe 4 | TACCCATTTGAGAACCTGTTGGCCC | HEX | BHQ-1 | Taqman probe for detection of *arcB2* transcript by qPCR |
| Probe 5 | AAGTTGCAGCGCATGATCAAGGTG | 6-FAM | BHQ-1 | Taqman probe for detection of *argF* transcript by qPCR |
| Probe 6 | AGAAGATCCTTATGCTGCGCCACC | HEX | BHQ-1 | Taqman probe for detection of *argGH* transcript by qPCR |
| Probe 7 | AGGTGTTATTTGGTATGATGCACA TGGTGA | 6-FAM | BHQ-1 | Taqman probe for detection of *rocF* transcript by qPCR |
| Probe 8 | TGGTAATCCACTTGCTTGTGCTGC | HEX | BHQ-1 | Taqman probe for detection of *rocD* transcript by qPCR |
| Probe 9 | AATCGGTGGCGACTTTGATCTAGC GAAAG | 6-FAM | BHQ-1 | Taqman probe for detection of *gyrB* transcript by qPCR. |
| Probe 10 | CCTGCAACAGCTTTCTTAACTTGC TCAC | HEX | BHQ-1 | Probe for detection of *tpiA* transcript by qPCR |
| Probe 11 | TGGGAAGAAGTACTAGGCACAAATTTGTGG | 6-FAM | BHQ-1 | Probe for detection of *ahrC* transcript by qPCR |

1. Projan, S.J. and G.L. Archer, *Mobilization of the relaxable Staphylococcus aureus plasmid pC221 by the conjugative plasmid pGO1 involves three pC221 loci.* J Bacteriol, 1989. **171**(4): p. 1841-5.

2. Kreiswirth, B.N., et al., *The toxic shock syndrome exotoxin structural gene is not detectably transmitted by a prophage.* Nature, 1983. **305**(5936): p. 709-12.

3. Fey, P.D., et al., *A genetic resource for rapid and comprehensive phenotype screening of nonessential Staphylococcus aureus genes.* MBio, 2013. **4**(1): p. e00537-12.

4. Halsey, C.R., et al., *Amino Acid Catabolism in Staphylococcus aureus and the Function of Carbon Catabolite Repression.* MBio, 2017. **8**(1).

5. Bose, J.L., *Genetic manipulation of staphylococci.* Methods Mol Biol, 2014. **1106**: p. 101-11.

6. Sharma-Kuinkel, B.K., et al., *The Staphylococcus aureus LytSR two-component regulatory system affects biofilm formation.* J Bacteriol, 2009. **191**(15): p. 4767-75.

7. Charpentier, E., et al., *Novel cassette-based shuttle vector system for gram-positive bacteria.* Appl Environ Microbiol, 2004. **70**(10): p. 6076-85.
